# Design and Implementation of Degenerate Microsatellite Primers for the Mammalian Clade

**DOI:** 10.1371/journal.pone.0029582

**Published:** 2011-12-27

**Authors:** Emmanuel Buschiazzo, Josephine S. Beck, Neil J. Gemmell

**Affiliations:** School of Biological Sciences, University of Canterbury, Christchurch, New Zealand; Smithsonian Institution National Zoological Park, United States of America

## Abstract

Microsatellites are popular genetic markers in molecular ecology, genetic mapping and forensics. Unfortunately, despite recent advances, the isolation of de novo polymorphic microsatellite loci often requires expensive and intensive groundwork. Primers developed for a focal species are commonly tested in a related, non-focal species of interest for the amplification of orthologous polymorphic loci; when successful, this approach significantly reduces cost and time of microsatellite development. However, transferability of polymorphic microsatellite loci decreases rapidly with increasing evolutionary distance, and this approach has shown its limits. Whole genome sequences represent an under-exploited resource to develop cross-species primers for microsatellites. Here we describe a three-step method that combines a novel in silico pipeline that we use to (1) identify conserved microsatellite loci from a multiple genome alignments, (2) design degenerate primer pairs, with (3) a simple PCR protocol used to implement these primers across species. Using this approach we developed a set of primers for the mammalian clade. We found 126,306 human microsatellites conserved in mammalian aligned sequences, and isolated 5,596 loci using criteria based on wide conservation. From a random subset of ∼1000 dinucleotide repeats, we designed degenerate primer pairs for 19 loci, of which five produced polymorphic fragments in up to 18 mammalian species, including the distinctly related marsupials and monotremes, groups that diverged from other mammals 120–160 million years ago. Using our method, many more cross-clade microsatellite loci can be harvested from the currently available genomic data, and this ability is set to improve exponentially as further genomes are sequenced.

## Introduction

Microsatellites, also called simple sequence repeats, consist of short (1–6 bp), tandemly repeated DNA motifs dispersed throughout genomes. Microsatellite sequences mutate through motif insertions and deletions along the repeat array, often at rates several orders of magnitude higher than the average genomic mutation rate [1]. Increasing numbers of polymorphic microsatellites are being associated with genetic disorders and variation in gene expression [2], but the high mutation rate at microsatellite loci also offers an abundant and readily available polymorphism that has been the foundation for the wide use of microsatellites as neutral molecular markers, especially in applications requiring fine temporal and/or spatial resolution, e.g. population genetics and forensics.

Despite a number of recognized advantages of microsatellites over other genetic markers, such as easy sample preparation and high information content [3], [4], the costs and time required to develop new polymorphic microsatellite markers can be prohibitive [5]. The recent decline in sequencing costs has paved the way for more efficient methods of *de novo* microsatellite isolation, but only when whole genome sequences [6] or large amounts of sequences are already available [7], [8] or purposely produced for the species of interest [9]; conditions that still imply a significant upstream investment.

Seeking to yield large amounts of genetic information with the least initial effort and cost, investigators commonly make attempts at transferring known microsatellite markers between species, typically from previously examined focal species to related non-focal species (e.g. [10], [11]; see [12] for a recent review). Successful transfer of microsatellite markers therefore requires (i) 1∶1 orthology of microsatellite loci, (ii) flanking sequences which are sufficiently conserved between species to provide PCR priming sites for cross-species amplification, and (iii) a microsatellite sequence which exhibits an appropriate level of polymorphism in the non-focal species. All three aspects are typically unknown at the onset of a project. Because there is a strong positive relationship between time of divergence and the accumulation of sequence differences along lineages [13], the consensus found in the literature that microsatellite transferability rapidly decreases with increasing distance between focal and non-focal species is not surprising [11], [12], [14]–[16].

With no prior focus on reducing the impact of these limitations, the traditional cross-species microsatellite transfer approach has had varying, generally disappointing, levels of success [12]. The use of databases of microsatellites located in expressed, thus putatively conserved genomic regions, have improved the expected and observed rate of cross-species transferability (e.g. [17]–[21]), especially with the complementary use of genome sequences from related species [22], [23]. However, multiple whole-genome alignments have not yet been exploited to date to explore and maximize the limits of microsatellite marker transferability.

Here, we present a novel and economic strategy that exploits our recent advances in building comprehensive datasets of microsatellites conserved across the mammalian clade [24]–[26]. We created a reproducible and adaptable framework that has allowed us to develop mammal-wide degenerate primers for nine dinucleotide microsatellites, five of which were successfully genotyped across most of a panel comprising 18 divergent species that represent the major mammalian orders, and three of which displayed high intraspecies polymorphism throughout the mammals tested. We conclude from this successful initial trial that this approach has much promise and paves the way for equivalent studies in other genera as the push towards obtaining genome sequences from multiple animal, predominantly vertebrate, species becomes a reality [27]. In addition, it provides a significant starting resource for those wishing to focus on specific mammalian species or groups of species where large numbers of microsatellite markers with robust cross-species utility are required.

## Materials and Methods

**Figure 1:**
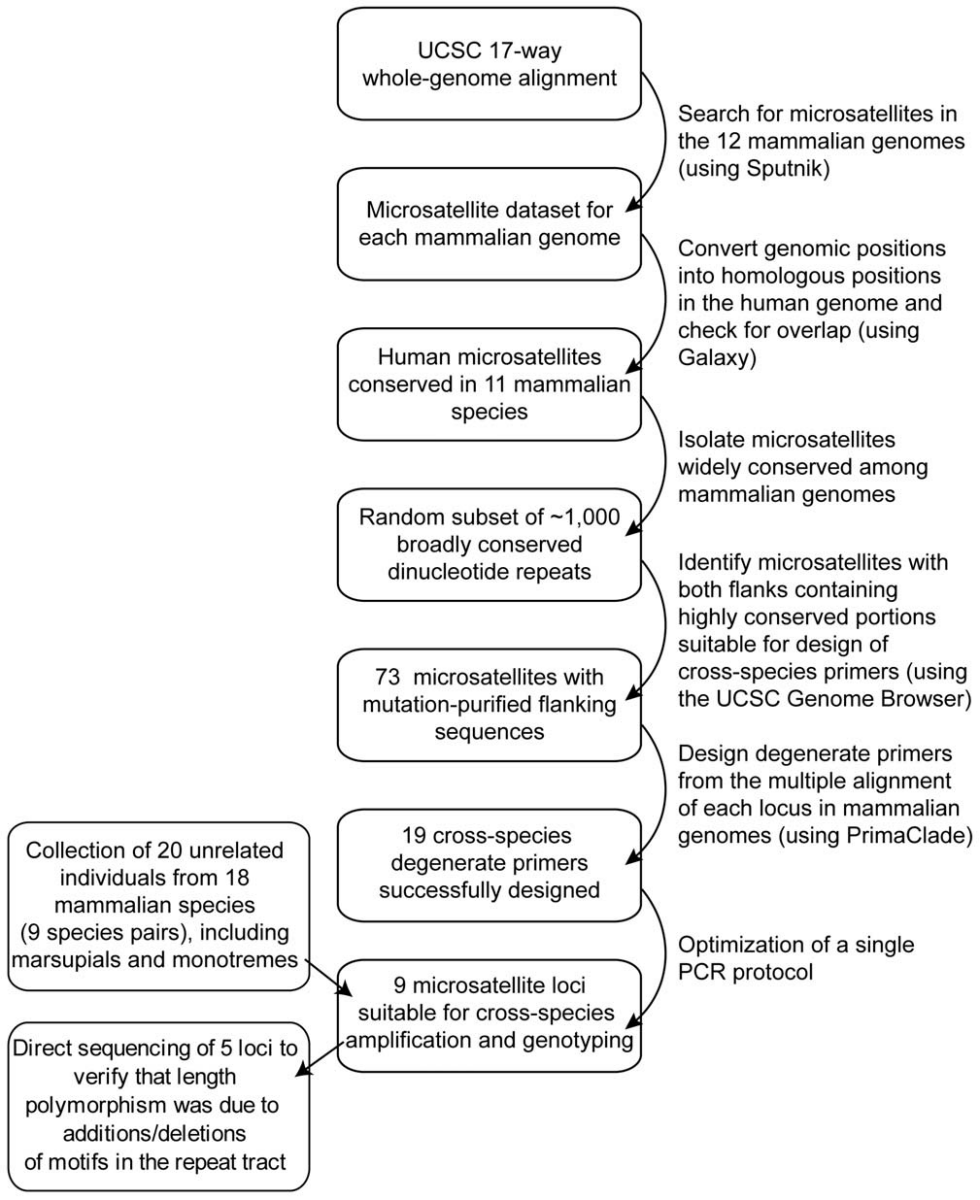
Schematic representation of the pipeline developed to design and implement degenerate cross-species primers for mammal-wide microsatellite loci. The University of California, Santa Cruz (UCSC) Genome Brower can be found at http://genome.ucsc.edu/.

Our overall strategy is presented in Figure 1.

### Ethics Statement

Information S1 shows the origin of our samples for each species included in this study. Restricted and general biological products (tissue or DNA) were imported with the New Zealand Ministry of Agriculture and Forestry (MAF) import permits No 2007031396 and 2007032360, respectively, issued for the University of Canterbury. Human DNA was sent from the National Cell Bank of Iran. Chimpanzee samples Pt163, Pt180, Pt203, and Pt254 were obtained as blood samples from the Iberia Research Center during routine veterinary care, and were processed in A. Stone's laboratory in Arizona State University, USA.

### Sample collection and DNA extraction

Mammalian species were chosen to include nine sister species pairs (n = 18) representing three of the four superorders of eutherians (Laurasatheria, Euarchontoglires and Afrotheria), as well the too often neglected marsupials and monotremes. We collected DNA, blood or tissue samples from 20 presumably unrelated individuals per species (Information S1). Total DNA was extracted using slight variations of the Chelex method [28], and quantified using an ND-1000 spectrophotometer (NanoDrop).

### *In silico* identification of conserved mammalian microsatellites

Orthologous mammalian microsatellites were identified using the UCSC vertebrate 17-WA [29] and a variation of an approach detailed elsewhere [26]. Briefly, FASTA-formatted sequences were extracted from the alignment in a pairwise fashion (human-other species) using Gmaj (http://globin.cse.psu.edu/dist/gmaj/), and the microsatellite search was carried out with a modified version of Sputnik [30], using the following parameters: –v 1 –u 5 –n -4 –s 8 –L 15 (motif length: 1–5 bp; mismatch penalty: -4; min score: 8, min array length: 15 bp). Each dataset was filtered for single-copy and repeat-free loci, and classified according to motif type, length, purity and complexity. Genomic positions of non-human microsatellites were converted to homologous positions in the human genome using a stand-alone version of Galaxy [31] and resources available at the UCSC Genome Browser (i.e. liftOver tool and conversion files). Conservation was assigned when genomic positions of human microsatellites overlapped with converted positions of non-human microsatellites.

### *In silico* isolation of potential cross-species microsatellite loci

An initial subset of ∼1,000 human dinucleotide microsatellites (length ≥14 bp) was randomly selected from a pool of broadly conserved microsatellites in the mammalian clade, i.e. present in at least in five mammals, or in comparisons including at least human, opossum and either dog or mouse. Mammalian species included in this study shared a common ancestor 160 MYA, and thus the chances of finding conserved *and* polymorphic were expected to be low. Although microsatellites composed of larger motifs, e.g. tri- and tetranucleotide repeats, are known to be less prone to genotyping errors than dinucleotide repeats, we chose to look and test the latter over the former because they tend to be longer and thus more polymorphic [32]. We did not require that microsatellites in this initial subset be conserved in all nine non-primate species for two reasons. First, most genome sequences in the 17-WA are incomplete, thus there is a non-negligible possibility for false negatives. Second, otherwise conserved microsatellites may be too short or overly interrupted to be detected using our *in silico* strategy in some genomes.

In order to optimize the identification of cross-species microsatellites with flanking sequences conserved across the entire mammalian clade, including monotremes (platypus), we reviewed by eye each microsatellite locus in the 28-way conservation track [33], an updated and enlarged version of the 17-WA (Information S2). Criteria for selection were: (i) presence of a dinucleotide repeat in all taxa included in our sample collection; although exceptions were tolerated for low-coverage (2×) genomes (cat, armadillo, elephant and tenrec), (ii) a relative extent of interspecies length variation in the repeat array, i.e. microsatellites with no or very limited length variation between species were discarded, (iii) ∼20 or more near-identical contiguous base pairs on both sides of the microsatellite sequence across all mammalian species, and (iv) total length of the potential amplicon not exceeding ∼400 bp. The purpose of this process, which by nature was relaxed because it was carried out by eye, was to filter out those microsatellites that did not meet the general requirements for cross-species markers, i.e. no variation in motif, but variation in length across species, conserved potential primer sites on both sides of the microsatellite and length of the amplicon compatible with current genotyping technology. We removed from these alignments any sequence derived from species not included in our sample collection, with the exception of sequences from armadillo (Xenarthra), elephant (Afrotheria) and opossum (Marsupialia), ensuring that each alignment covered the entire breadth of the Mammalia. When necessary, microsatellite flanking sequences were re-aligned manually using BioEdit [34].

### *In silico* comparative primer design

Alignments were submitted to PrimaClade [35]; this web application runs Primer3 [36] independently for each sequence, collating the results to identify primers that bind across the alignment, while allowing for base degeneracy. A maximum of three degenerate sites per primer were allowed. Primers that overlapped gaps (indels) in the alignment were excluded, and only primers generating fragments smaller than 350 bp were kept for further study. Using the Java web-application NetPrimer and the developer's recommendations (PREMIER Biosoft International, http://www.premierbiosoft.com/netprimer/), potential primer pairs were tested for the presence of secondary structures (hairpins, self- and cross-dimerization), palindromes and repeats that could affect the amplification reaction through intra- and intermolecular interactions and non-specific annealing. Table 1 summarizes the overall set of unambiguous criteria that were applied to increase chances of successful amplification and select the optimal cross-species primer pair at each locus. In addition, the same criteria were used to design primers for a locus containing the non-coding microsatellite with the widest range of conservation in mammals described to date, and located in the 3′-UTR of the NCAM1 gene [37]. A list of all degenerate cross-species primers and their characteristics is displayed in Information S3.

**Table 1:** Selection criteria for designing comparative microsatellite primers.

					Repeats		Stability of primer secondary structures (ΔG*)					
L_expected_	L_primer_ †	T_m_ ‡	ΔT_m_	%GC§	2-6×	1×	3′ HP	Int HP	3′ SD	Int SD	3′ CD	Int CD
<350	18–22	58–62	<1	45–60	<3	<6	>−2.00	>−3.00	>−5.00	>−6.00	>−5.00	>−6.00

L_expected_: expected length of PCR products (bp); L_primer_: primer length (bp); Tm: melting temperature (°C); ΔT_m_: T_m_ difference between both primers; %GC: G+C content; 2-6×: number of tandemly repeated non-mononucleotide motifs (2–6 bp); 1×: length of mononucleotide runs; ΔG: Gibbs free energy required to break the secondary structure (kcal/mol); 3′: 3′-end of primers; Int: Internal; HP: hairpin, SD: self-dimer, CD: cross-dimer.

* Output from NetPrimer; criteria as recommended in the application's manual.

† Exceptionally up to 26 bp.

‡ Output from PrimaClade.

§ A 30–62% range was tolerated for primers >22 bp.

### DNA amplification, genotyping and sequencing

We followed the M13-tail PCR method of [38] and optimized it for cross-species investigation. Amplifications were performed on a Master Cycler Gradient (Eppendorf), in 15 µl of reactions containing 0.66 µM of reverse-specific primer, 0.66 µM of fluorescent dye-labelled M13 primer, 0.33 µM of forward-specific primer with M13-tail, 2.5 mM of MgCl_2_, 0.2 mM of each dNTP, 4 mM of tetramethylammonium chloride (TMAC), 0.75 U of BioTaq DNA polymerase (Bioline), and 20–100 ng of genomic DNA template. A touch-down PCR was undertaken in which the initial annealing temperature T_init_ (generally 59°C, but see exceptions in Information S3) was reduced at the rate of 2°C every two PCR cycles until the target temperature (T_targ_ = T_init_−10°C) was reached; 26 regular cycles were then performed at T_targ_. The general thermocycling profile was as follows: initial denaturation at 94°C for 3 minutes; denaturation at 94°C for 15 s, annealing for 30 s, extension at 72°C for 20 s; final extension at 72°C for 20 minutes. PCR efficiency was assessed through electrophoresis of 3 µl of amplified products loaded on 1.5% agarose gel stained with BET. Primer pairs resulting in multiple bands or no amplification in all or most species were discarded. Fragment analysis was performed in an ABI3100 Genetic Analyzer (Applied Biosystems) following instructions from the manufacturer. Fragment sizes were scored with GeneMarker (Soft Genetics LLC). Expected and observed heterozygosities and polymorphic information content (PIC) were measured for all genotyped loci in each species with CERVUS 3.0.3 [39], [40]. We restricted sequencing to the loci where genotyping was successful in a broad range of species. Four individuals per species per locus were selected for direct sequencing on a locus per locus basis based on homozygozity and, where possible, polymorphism. The sequencing PCR was run using a standard protocol (Big Dye Terminator Cycle Sequencing Kit, Applied Biosystems), and products were prepared for sequencing in both directions in an ABI3100 Genetic Analyzer (Applied Biosystems) following manufacturer's instructions. Sequences obtained for each locus were aligned with ClustalW [41], and edited manually using BioEdit [34].

## Results

### Candidate cross-species microsatellite markers for the mammalian clade

A total of 126,306 human microsatellites were found conserved in at least one of the non-primate mammalian species, i.e. in mouse, rat, rabbit, dog, cow, elephant, armadillo, tenrec and/or opossum (Information S4). An initial subset of ∼1,000 human dinucleotide microsatellites (length ≥14 bp) was randomly selected from a total pool of 5,596 microsatellites (including 2,756 dinucleotide repeats) that were broadly conserved across aligned genomes. Furthermore, a total of 73 28-WA intervals, each comprising a potential mammal-wide microsatellite locus, were selected for the presence of a polymorphic dinucleotide microsatellite flanked by stretches of ultra-conserved sequences potentially suitable for cross-species primer design (Information S2).

Degenerate primer pairs were then successfully designed for 19 microsatellite loci. Of those 19 primer pairs tested using a unique, optimized set of PCR conditions, nine pairs yielded a scorable band pattern in all tested mammalian samples (Information S5). There was no significant difference in amplification success between highly and slightly degenerate primer pairs, nor did primer G+C content of sequence affect amplification success (Information S3).

### Intraspecies polymorphism

To test our set of mammal-wide microsatellite loci for length polymorphism at the population level, amplicons were produced and genotyped in each 20-sample set. Of nine primer pairs developed for cross-species genotyping, five were successful in providing allele length data at the population level across most species. Table 2 shows allelic richness and estimates of heterozygosities (expected and observed) for each locus in each species, whenever genotyping was successful; Information S6 shows polymorphic information content (PIC). Although these values should be considered rough guides given the limited sample sizes, three microsatellite loci showed significantly more intraspecies polymorphism (C2-6868, C2-1915 and C2-1218), indicating potential suitability for marker-based applications across the mammalian clade.

**Table 2:** Polymorphism at nine cross-species mammalian microsatellite loci.

	C2-1218*	C2-6868*	C2-1915*	C4-1514*	C6-1112	C9-1918	C14-9692	C15-3531	C17-4243*
Human	268–294 (9/18)	228 (1/20)	166–178 (5/17)	281–283 (2/20)	152–156 (2/19)	300–302 (2/14)	234–237 (3/20)	226–228 (2/17)	311 (1/20)
	0.47/0.82	0/0	0.71/0.64	0.30/0.26	0.11/0.19	0.38/0.52	0.05/0.15	0.29/0.26	0/0
Chimpanzee	n/a	n/a	n/a	n/a	n/a	n/a	n/a	n/a	n/a
Mouse	291–301 (10/20)	242–291 (16/17)	216–238 (10/14)	313–317 (4/19)	155–161 (3/18)	311 (1/20)	240–242 (2/19)	297–299 (2/16)	319–325 (5/19)
	0.30/0.76	0.56/0.93	0.50/0.91	0.22/0.52	0.50/0.51	0/0	0.26/0.56	0.13/0.12	0.42/0.62
Rat	274–280 (2/20)	236 (1/20)	176 –180 (3/20)	274 (1/20)	158–162 (3/19)	n/a	n/a	237 (1/20)	326 (1/15)
	0.45/0.36	0/0	0.35/0.31	0/0	0.47/0.55			0/0	0/0
Dog	268–278 (9/20)	256–268 (4/18)	180–191 (5/19)	297–299 (2/17)	3 peaks (1/11)	n/a	214 (1/20)	n/a	309 (1/20)
	0.40/0.83	0.28/0.52	0.47/0.78	0.12/0.11			0/0		0/0
Cat	265–276 (8/20)	n/a	176–188 (6/19)	n/a	n/a	n/a	n/a	n/a	312 (1/20)
	0.75/0.82		0.63/0.72						0/0
Cow	259–264 (2/18)	231 (1/20)	167–169 (2/20)	281 (1/20)	146 (1/20)	300–305 (2/20)	208 (1/20)	240–242 (2/20)	308 (1/20)
	0.06/0.06	0/0	0.15/0.22	0/0	0/0	0.50/0.51	0/0	0.05/0.05	0/0
Sheep	270–280 (8/19)	229–237 (4/14)	163–173 (4/15)	292 (1/20)	146 (1/20)	307–308 (2/20)	208–212 (3/18)	n/a	306 (1/20)
	0.58/0.83	0.29/0.37	0.56/0.64	0/0	0/0	0/0.10	0.39/0.60		0/0
Dolphin	264–278 (4/19)	n/a	160–176 (7/19)	291–295 (2/16)	148–150 (2/19)	313–319 (3/16)	214–215 (2/19)	226 (1/20)	303–304 (2/20)
	0.47/0.61		0.74/0.81	0.13/0.12	0.17/0.25	0.50/0.59	0/0.27	0/0	0/0.39
Pilot Whale	265 (1/20)	243 (1/20)	161–174 (6/17)	292 (1/19)	148 (1/20)	313–317 (4/18)	216 (1/20)	223 (1/20)	307 (1/20)
	0/0	0/0	0.82/0.79	0/0	0/0	0.39/0.70	0/0	0/0	0/0
Hedgehog	260–272 (5/20)	225–230 (5/20)	268–172 (3/20)	321–325 (2/20)	148–154 (2/20)	345 (1/20)	151–157 (2/20)	213–227 (7/20)	303 (1/20)
	0.65/0.70	0/0.10	0.40/0.56	0.45/0.48	0.25/0.30	0/0	0.25/0.30	0.65/0.81	0/0
Shrew	309–329 (11/20)	254–256 (3/20)	221–223 (2/20)	281 (1/20)	n/a	n/a	n/a	n/a	309–313 (4/19)
	0.80/0.88	0.50/0.45	0/0	0/0					0.26/0.25
Dugong	269–273 (4/20)	225 (1/17)	176 (1/20)	274 (1/19)	138 (1/17)	289 (1/18)	n/a	222 (1/17)	294–298 (3/18)
	0.45/0.57	0.05/0.05	0/0	0/0	0/0	0/0		0/0	0.50/0.41
Tenrec	n/a	n/a	n/a	281 (1/20)	n/a	n/a	n/a	n/a	316–319 (4/15)
				0/0					0.33/0.55
Tammar wallaby	249–291 (9/16)	n/a	193–195 (2/16)	281 (1/16)	149 (1/16)	n/a	n/a	191–293 (14/15)	325–332 (5/10)
	0.79/0.83		0.06/0.06	0/0	0/0			0.60/0.94	0.20/0.70
Quoll	241–243 (2/9)	318–342 (6/8)	n/a	297 (1/20)	148 (1/20)	n/a	203 (1/14)	n/a	299 (1/15)
	0.11/0.11/0.10	0.50/0.81/0.72		0/0/0	0/0/0		0/0/0		0/0/0
Platypus	245–263 (2/15)	346–382 (7/13)	214–226 (4/15)	n/a	145 (1/11)	n/a	208 (1/18)	n/a	298 (1/15)
	0/0.13/0.12	0.85/0.72/0.64	0.13/0.36/0.32		0/0/0		0/0/0		0/0/0
Echidna	248–252 (4/15)	372–376 (4/13)	n/a	317 (1/14)	142 (1/12)	278 (1/20)	205–213 (5/14)	194–196 (2/12)	298 (1/17)
	0.20/0.57	0/0.65		0/0	0/0	0/0	0.50/0.76	0.09/0.09	0/0

Allelic Range (number of alleles/number of individuals successfully genotyped) Observed Heterozygosity/Expected Heterozygosity.

* indicates sequenced loci.

It is interesting to weigh the extent of polymorphism at each locus against the sequence data that is available from the 28-way alignments. Indeed, intraspecies polymorphism is largely influenced by the length of pure repeat segments within the microsatellite sequence, with long pure microsatellite tracts tending to be more polymorphic than short and/or degenerated microsatellites [1]. Accordingly, the highly polymorphic C2-1218 locus contained long pure tracts of (CA) motifs in most species used for genotyping (Information S7). The C2-6868 locus showed less variability and contained many sub-units of short size (<8 repeats), with the exception of a long and extensively polymorphic tract in mouse (Information S7). Despite imperfections in the microsatellite sequence, the widely polymorphic C2-1915 locus generally contained at least one long pure sub-unit, i.e. >8 repeats (Information S7). The two other loci, C2-1514 and C17-4243, showed less polymorphism and generally contained short tracts (Information S7). Against expectations, we observed a few exceptionally long tracts with no intraspecies variability in allele length, for example the C17-4243 locus in rat. This may be explained by unintended close relatedness of individuals among some sample sets, e.g. rats and pilot whales (discussed below).

### Relationship between changes in flanking sequences and locus length

Although sequencing is not standard practice in most applications of microsatellite markers, we sought to examine in detail the relationship between DNA sequence and the nature and extent of polymorphism of our most successful cross-species microsatellite loci across the studied species. Sequence-level information is indeed essential to inspect (i) whether allele length variations are attributable to additions/deletions of motifs within microsatellite sequences rather than indels in the flanking sequences, (ii) what is the extent of size homoplasy, if any, among alleles (homoplastic alleles have identical length but different sequence), (iii) the relationship between microsatellite structure and polymorphism [42]–[44]. Ideally, a microsatellite marker exhibits polymorphism through addition/removal of repeats only, has no or a non-significant fraction of homoplastic alleles, and has a simple repeat structure with mutational dynamics in line with current models of microsatellite evolution [45], [46].

We carried out cross-species direct PCR sequencing of the five most successfully genotyped microsatellite loci, namely C2-1218, C2-1915, C4-1514, C9-1918 and C17-4243 (Table 2). Four homozygous allele variants (where available) were sequenced for each species. Information S8 presents an overview of these results, with total fragment length, microsatellite length and microsatellite sequence given for all variants of successfully sequenced individuals. The direct PCR sequencing success rate was average (42%), regardless of previous genotyping success. Of 56 cases where between two to four sequences per locus per species could be retrieved, we found 26 cases of intraspecific polymorphism (i.e. length and/or sequence polymorphism), with a total of 31 new intraspecific alleles (36 if we include chimpanzee, for which we have no genotyping data). Here we define a ‘new allele’ as an allelic variant of an arbitrary ‘ancestral’ allele (Information S8).

Ten out of the 31 new allele variants showed a difference between total length change and microsatellite length change. These differences are most likely the result of short indels occurring in flanking regions. However, we cannot be certain for all cases, due to the absence of flanking sequence information, and because genotyping errors cannot be completely ruled out. In the other 21 comparisons (68%), changes in total locus length were consistent with repeat addition or removal in the microsatellite sequence. Although six cases of size homoplasy were observed (identical size, different sequence), only two originated from mutations in both microsatellite and flanking sequences, the other four cases originating from a point mutation within the microsatellite sequence. Finally, in all cases, addition/removal of one or more motifs occurred in the longest pure tract(s) of dinucleotide repeats.

## Discussion

Microsatellites are currently one of the most popular types of genetic markers for molecular ecology, forensics and genome mapping studies. Their evolutionary dynamics have been extensively studied [1], [3], [45], and new analytical approaches are continually being developed [47], [48]. However, their use could be facilitated, and even extended, if microsatellite markers could be readily transferred between species. Most attempts to transfer microsatellites across species are hindered by the accumulation of point mutations in microsatellite flanking sequences and/or the decay of microsatellite sequences over time [1], [12]. But the recent finding that scores of microsatellite loci are indeed conserved across vertebrate genomes [25], [26] has offered new hopes of significantly increasing success rates in developing cross-species microsatellite markers than have been observed to date [12].

Here we described a novel combination of *in silico* and wet-lab approaches to develop a set of microsatellite markers with broad, potentially universal, utility across the Mammalia (Figure 1). We demonstrated that an easily adaptable and reproducible protocol can be used to extract highly conserved microsatellite loci from multiple genome alignments, design degenerate primers and implement a set of microsatellite loci across vastly distant species – in this case 18 mammalian species that shared a common ancestor no earlier than 160 million years ago [24]. Although there are anecdotal reports of exceptional conservation in other taxa, e.g. turtles [49] and fish [50], this extensive transferability exceeds that of any prior cross-species study in mammals, and thus radically alters the conventional assumption that cross-species amplification of microsatellite loci is limited to closely related species [12].

Focusing our analysis on the entire breadth of the Mammalia (eutherians, marsupials and monotremes) ensured a large evolutionary scope as well as a solid genomic framework where scores of conserved microsatellites have been identified [26]. Given the extensive species divergence, it was expected that only a small fraction, if any, of the subset of widely conserved loci would not only provide a substrate to develop mammal-wide PCR primers, but also contain a polymorphic microsatellite sequence in all genomes. Our investigation shows that contrary to this common expectation, mammalian genomes contain a significant number of potential mammal-wide microsatellite markers. First, the proportion of microsatellite loci found to contain potential conserved primer sites in a first non-stringent *in silico* scan was fairly high, with conserved primer sites identified in 7.3% of the random subset of ∼1,000 conserved dinucleotide repeats that we drew from our total pool of 5,596 highly conserved microsatellite loci. From those 1,000 conserved dinucleotide repeats, 19 or 1.9% were suitable to design mammal-wide degenerate primers using our stringent set of criteria (Table 1), result we view as remarkable considering the breadth of the Mammalia and the limited number of sites that we studied. Indeed, our initial subset represented only a fraction, less than a fifth, of all the microsatellites found that could be examined to identify cross-species microsatellite markers. In addition, using a more comprehensive dataset of conserved mammalian microsatellites [26], we were able to find 4,084 human dinucleotide repeats among 10,267 conserved microsatellites in five non-primate mammals. Thus, by extrapolation at least 80 loci should be suitable for primer design using these selection criteria, and we anticipate that more should be identified under less stringent conditions (e.g. conservation in human-mouse-opossum). Moreover, other types than dinucleotide repeats can also be used for cross-species transfer of microsatellite markers, e.g. tetranucleotide markers, which are conserved in equivalent numbers in mammalian genomes [26]. Furthermore, if there is success in designing comparative primers useful across the Mammalia, then many more are expected to be developed from more specific comparisons, i.e. within subgroups of the Mammalia, especially with further genomic resources being acquired [27].

There was no particular relationship between PCR success and either G+C-content of PCR priming sites, genomic location, and number of degeneracy in primer sequences (Information S3). Of 19 designed primer pairs, nine were successfully optimized for mammal-wide amplification, and five were suitable for genotyping and sequencing. A number of methodological choices were made to decrease costs, but they may have reduced success rates in genotyping and sequencing, e.g. Chelex extraction method (impure DNA extract), M13-genotyping (primer dimers, inconsistent fluorescent signal), use of degenerate primers (low amplification), and direct PCR sequencing (low quality reads). We would expect a significant increase in success rate using clean extraction methods (extraction kit, phenol-chloroform protocol), standard fluorescent genotyping, non-degenerate primers and clone sequencing. In addition, we had little or no control on sampling and DNA quality for most of our samples, which may have had detrimental consequences on the overall quality of our results. For example, low polymorphism in rats and pilot whales could be explained by our samples originating from inbred populations [51] and pod strandings, respectively [52]. Drawing on these experiences, guidelines are outlined in the supplementary materials to help others planning to use conserved microsatellites to develop comparative primers (Information S9).

Overall, our cross-species primers still yielded good genotyping results for five of the nine fully optimized loci. Intraspecies polymorphism was strongly associated with length and purity of repeat tracts, which emphasized the importance of examining the sequence structure of microsatellites to select polymorphic genetic markers. Sequence information demonstrated that most changes (68%) in total fragment length at the five loci were attributable to mutations in the microsatellite sequence rather than in the flanking sequences, suggesting that cross-species primers designed for these loci are invaluable candidates for being employed as universal genetic markers across the Mammalia, as it has already been demonstrated for the under-studied short-beaked echidna [8].

Our findings establish a new paradigm in that they demonstrate that with the emergence of large numbers of genome sequences for a given taxonomic group, universal sets of microsatellite markers can be generated for that group, using a simple protocol. Provided that such sets are fully characterized and tested for confounding influences in the the different species of interest (e.g. linkage and deviations from the Hardy-Weinberg equilibrium), and standardized for use in different laboratories, this creates the genuine possibility of developing large panels of microsatellites with cross-species transferability and known genomic context [16], enabling true inter-study comparability that have long been sought but never before obtained.

## Supporting Information

Information S1 **Nature and origin of mammalian samples.**
(PDF)

Information S2 **28-way alignment of conserved microsatellites.** Both flanking sequences of the conserved microsatellite (represented by the black bar) contain a stretch of orthologous sequences potentially suitable to design cross-species primers (indicated with orange boxes). Locus C2-1218 at chr2:17,699,950–17,700,450 (UCSC hg18).
(PDF)

Information S3 **Description of 19 primer pairs selected for PCR optimization.**
(PDF)

Information S4 **Distribution of human microsatellites conserved in nine non-primate species.** The human data (in bold) correspond to the total number of human microsatellites found to be conserved in at least one species. Species-specific subsets correspond to the number of human microsatellites that are conserved in at least each one of those species. Numbers in brackets indicate numbers for the whole genome (excluding Y chromosome).
(PDF)

Information S5 **Electrophoresis of PCR products for C2-1218 in 17 mammals.** *Bta*: cow, *Oar*: sheep, *Cfa*: dog, *Mmu*: mouse, *Fca*: cat, *Meu*: tammar wallaby, *Oan*: platypus, *Gme*: pilot whale, *Dma*: quoll, *Rno*: rat, *Ddu*: dugong, *Sar*: shrew, *Eeu*: hedgehog, *Tac*: echidna, *Hsa*: human, *Tad*: dolphin, *Ete*: tenrec. (negative control: water).
(PDF)

Information S6 **Polymorphic Information Content (PIC) at nine cross-species microsatellite loci based on at most 20 individuals per species.**
(PDF)

Information S7 **UCSC 28-way alignment of five cross-species microsatellite locus showing species of interest (in order: C2-1218; C2-6868; C2-1915; C4-1514; C17-4243).** (A) Flanking sequences. Underscores represent the microsatellite sequence; positions are counted upstream and downstream from the microsatellite. Boxes indicate primer sites, dashes gaps and dots bases identical to human. (B) Microsatellite sequence. Array length is shown in brackets. UCSC assemblies: Human (hg18), chimp (panTro2), mouse (mm8), rat (rn4), cow (bosTau3), dog (canFam2), cat (felCat3), shrew (sorAra1), hedgehog (eriEur1), armadillo (dasNov1), elephant (loxAfr1), tenrec (echTel1), opossum (monDom4), platypus (ornAna1).
(PDF)

Information S8 **Allele length, and microsatellite length and sequence variation in at most four individuals for five cross-species microsatellite loci.** L_allele_: allele length (bp); L_micro_: microsatellite length (bp); an individual (Indiv 1) 1 is used as a reference to measure length variation (+/−).
(PDF)

Information S9 **Guidelines to facilitate the identification, design, optimization and implementation of comparative microsatellite primers.**
(PDF)
