# Supplementary material for: Design and Implementation of Degenerate Microsatellite Primers for the Mammalian Clade
Source: PLoS One. 2011 Dec 27;6(12):e29582. doi: 10.1371/journal.pone.0029582 (PMC3246486; doi:10.1371/journal.pone.0029582)
Supplement: Information S1 — Nature and origin of mammalian samples. (PDF) [file pone.0029582.s001.pdf]

Supporting Information 1: Nature and origin of mammalian samples

Superorder	Order	Common name	Scientific name	Seq*	CITES†	#‡	Type	Contact	Institution
Laurasiatheria	Cetartiodactyla	Cow	*Bos taurus*	Y	-	20	DNA	J. Hickford	Lincoln U, New Zealand
		Sheep	*Ovis aries*	Y	-	20	DNA	J. Hickford	Lincoln U, NZ
		Dolphin	*Tursiops aduncus*	N	-	20	Tissue	L. Moller	Macquarie U, Australia
		Pilot whale	*Globicephala melas*	N	II	20	Tissue	M. Oremus, S. Baker	Auckland U, NZ
	Carnivora	Cat	*Felis catus*	Y	-	20	Blood	-	Gribbles Veterinary, NZ
		Dog	*Canis familiaris*	Y	-	20	Blood	I. Vargas-Jentzsch	U of Canterbury, NZ
	Eulipotyphla	Hedgehog	*Erinaceus europaeus*	Y	-	20	DNA	M. Hale	U of Canterbury, NZ
		Shrew	*Sorex araneus*	Y	-	20	Tissue	G. Yannic, J. Hausser	U of Lausanne, Switzerland
Euarchontoglires	Rodentia	Mouse	*Mus musculus*	Y	-	20	DNA	D. Tautz	Köln U, Germany
		Rat	*Rattus norvegicus*	Y	-	20	Tissue	B. Robertson	U of Canterbury, NZ
	Primates	Human	*Homo sapiens*	Y	-	20	DNA	A. Amanzadeh, F. Shokri	National Cell Bank of Iran
		Chimpanzee	*Pan troglytes*	Y	II	20	Outsourced	A. Stone	Arizona State U, USA
Afrotheria	Afrosoricida	Tenrec	*Echinops telfairi*	Y	-	20	Tissue	S. Goodman	Field Museum, Chicago, USA
	Sirenia	Dugong	*Dugong dugong*	N	I	20	DNA	A. MacMahon, D. Blair	James Cook U, Australia
Australidelphia	Diprotodontia	Tammar wallaby	*Macropus eugenii*	Y	-	16	DNA	J. Graves	Australian National U, Australia
	Dasyuridae	Quoll	*Dasyurus maculatus*	N	-	20	Tissue	M. Cardoso	U of New South Wales, Australia
n/a	Monotremata	Platypus	*Ornithorhynchus anatinus*	Y	-	20	DNA	C. Whittington	U of Sydney, Australia
		Echidna	*Tachyglossus aculeatus*	N	-	20	DNA/tissue	S. Nicol	U of Tasmania, Australia
								P. Rismiller	Kangaroo Island, Australia

* indicates whether genome sequence was partly or completely available at the time of the study
† CITES Appendices for listed protected species (http://www.cites.org/eng/resources/species.html)
‡ Number of sourced individual samples
