# Supplementary material for: Design and Implementation of Degenerate Microsatellite Primers for the Mammalian Clade
Source: PLoS One. 2011 Dec 27;6(12):e29582. doi: 10.1371/journal.pone.0029582 (PMC3246486; doi:10.1371/journal.pone.0029582)
Supplement: Information S2 — 28-way alignment of conserved microsatellites. Both flanking sequences of the conserved microsatellite (represented by the black bar) contain a stretch of orthologous sequences potentially suitable to design cross-species primers (indicated with orange boxes). Locus C2-1218 at chr2:17,699,950–17,700,450 (UCSC hg18). (PDF) [file pone.0029582.s002.pdf]

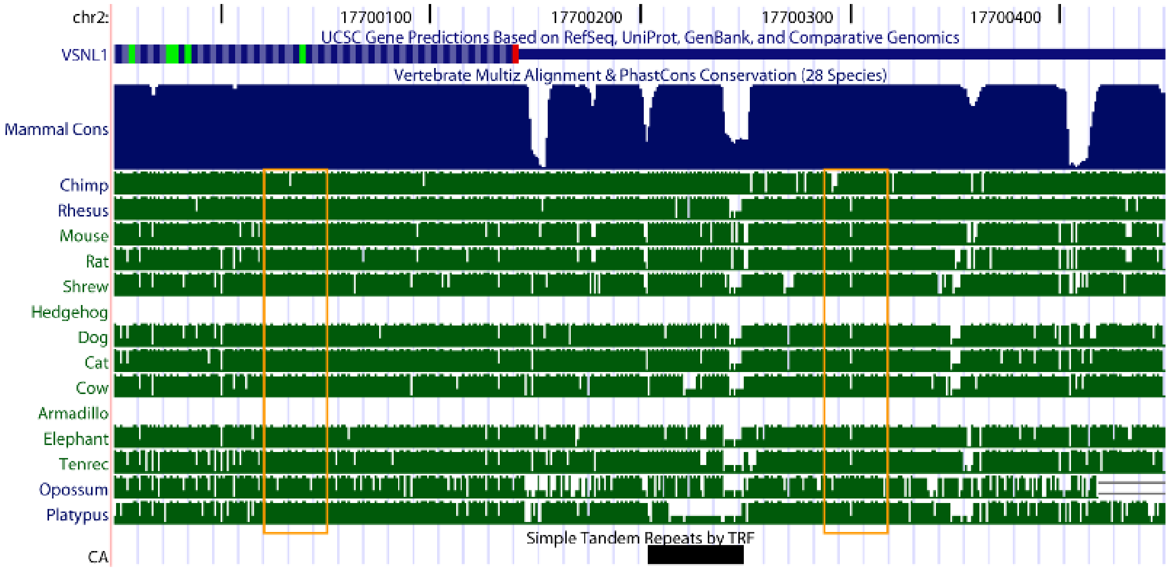

Supporting Information 2: 28-way alignment of conserved microsatellites. Both flanking sequences of the conserved microsatellite (represented by the black bar) contain a stretch of orthologous sequences potentially suitable to design cross-species primers (indicated with orange boxes). Locus C2-1218 at chr2:17,699,950-17,700,450 (UCSC hg18).
