# Supplementary material for: Design and Implementation of Degenerate Microsatellite Primers for the Mammalian Clade
Source: PLoS One. 2011 Dec 27;6(12):e29582. doi: 10.1371/journal.pone.0029582 (PMC3246486; doi:10.1371/journal.pone.0029582)
Supplement: Information S3 — Description of 19 primer pairs selected for PCR optimization. (PDF) [file pone.0029582.s003.pdf]

Supporting Information 3: Description of 19 primer pairs selected for PCR optimization.

			DS					Expected fragment length (M13 excluded)													
Name	Sequence	L_{prim}	2^{ary}	3^{ary}	%GC	T_m	T_{init}	*Hsa*	*Ptr*	*Mmu*	*Rno*	*Cfa*	*Fca*	*Bta*	*Sar*	*Eeu*	*Ete*	*Meu*	*Oan*	Location (Gene)	Genotype	Seq
C1-2125	F=TCAGRGACTGGACCTTAGAAGA<br>R=AGRCTGCTTTTAGACCGTACC	22<br>21	1<br>1	-<br>-	50.00<br>52.38	60.24<br>59.80	59	133	133	143	137	131	145	133	139	135	?	?	276	3'-UTR (ZNF238)		
C2-1218	F=GGAYAAGAACAAAGAYGACCA<br>R=TGAMATTCTCATGCACACRAGT	21<br>19	2<br>2	-<br>-	47.62<br>40.91	60.14<br>59.22	60	281	276	295	285	272	273	267	316	**266**	266	?	248	3'-UTR (VSLN1)	Y	Y
C2-6868	F=CTTCCTCCAGAAGCCTCCTT<br>R=TGGTGRGTAAATGAATTAGACATGC	20<br>25	-<br>1	-<br>-	55.00<br>36.00	59.95<br>59.47	59	233	233	273	240	**264**	?	233	255	234	276	?	357	3'-UTR (MEIS1)	Y	Y
C2-1915	F=TCTTCTTCTTAATCACAATTCAGYC<br>R=CAAACAGCSMCAAACACC	25<br>18	1<br>2	-<br>-	36.00<br>55.56	60.14<br>59.94	59	174	160	197	176	184	?	173	228	172	?	**201**	223	Intron (ZEB2)	Y	
C3-1615	F=TGCTGTCTGGTAGRAGGGCT<br>R=TTTCCTGCTCYYGATGTT	20<br>18	1<br>2	-<br>-	55.00<br>50.00	60.01<br>59.17	59	233	231	236	235	244	244	233	233	223	221	?	?	Intron (ZBTB20)		
C4-1514	F=GGCATGTAWGTGGTTTTTGAACT<br>R=GATCTGGAACACTGAAGACKC	23<br>21	1<br>1	-<br>-	39.13<br>52.38	60.16<br>59.87	58	282	283	314	275	291	304	278	**322**	322	**280**	?	225	Intron (LCORL)	Y	Y
C5-1211	F=ACKTGGCAGCAGACCCTGT<br>R=TGGATCACATGGRGAAGC	19<br>18	1<br>1	-<br>-	57.89<br>55.56	60.88<br>60.43	59	269	269	280	280	258	**266**	260	259	261	263	?	260	3'-UTR (ZNF608)		
C6-5453	F=TTAGTMAAACCATTTGCGACY<br>R=TGGGCGTGRGGCTGWRCT	21<br>18	2<br>3	-<br>-	42.86<br>61.11	59.99<br>60.43	59	275	271	261	**259**	266	273	**252**	?	268	?	**244**	273	5'-UTR (NRN1)		
C6-1112	F=AATTGCTGYGCTAATTACAACAT<br>R=GACTTCTCCCAWGGCATVA	23<br>19	1<br>1	-<br>1	34.78<br>52.63	60.06<br>59.57	59	153	151	160	162	151	186	147	?	155	144	**150**	146	IGR (MRDS1)	Y	
C9-1918	F=GCCTTGCCAYGACCAYTT<br>R=GCTCYSCGCTCAATTTAATT	18<br>20	2<br>2	-<br>-	50.00<br>45.00	59.46<br>60.41	59	302	313	303	296	301	**330**	307	?	311	323	?	293	IGR (PBX3)	Y	Y
C11-1417	F=GGCAARAGACACTAGAGVARTG<br>R=RGYCMAGACGGTCCCCAA	22<br>18	2<br>3	1<br>-	50.00<br>55.56	59.15<br>59.02	59	207	218	228	225	179	203	197	?	204	184	**228**	?	3'-UTR (NCAM1)		
C13-7574	F=AATCAATATGGAAGGAGCGGT<br>R=HGCYTTTGTTTCAAGATGGT	21<br>20	-<br>1	-<br>1	45.00<br>42.86	59.81<br>60.11	59	253	255	275	303	254	257	254	253	247	267	**327**	265	Intron (BX647243)		
C14-2527	F=GGATGCTGAAATGACAGGC<br>R=GGTCTTCACACCAGGGRA	19<br>18	-<br>1	-<br>-	52.63<br>61.11	59.19<br>59.42	59	249	251	236	266	262	?	267	245	?	294	?	?	IGR (FOXG1B)		
C14-9692	F=TTAAGTGATTTTTGTAATGGTCCG<br>R=YACCTCCAACAACTTCCTGTAAT	24<br>23	-<br>1	-<br>-	33.33<br>43.48	59.35<br>59.44	59	234	227	234	239	215	**222**	209	?	?	239	**205**	210	IGR (BC043585)	Y	
C15-3531	F=CCAAGAGGGRTTATTTATGGC<br>R=TGATTTGACACTGKGARGG	21<br>19	1<br>2	-<br>-	47.62<br>52.63	59.69<br>59.46	59	228	226	298	224	240	256	244	265	223	?	**239**	195	Intron (BMF)	Y	
C17-4243	F=TGCMKAGGTTGCAGAATA<br>R=TRTCACATTTTACGACAATARCATTA	18<br>26	2<br>2	-<br>-	50.00<br>30.77	58.89<br>59.87	59	311	313	311	317	307	?	306	314	**305**	325	?	299	Intron (HOXB3)	Y	Y
C19-3338	F=GCTGTCCACCRAGATTCACC<br>R=GCATYCCTGCCTTTRACTAA	20<br>20	1<br>2	-<br>-	55.00<br>50.00	60.52<br>60.21	59	206	204	197	232	260	?	**196**	226	?	197	**170**	?	IGR (ZNF536)		
C20-3430	F=GGAAGAATGGCGTAATGCTG<br>R=CGTTTCCAATTCCYGAGTT	20<br>19	-<br>1	-<br>-	50.00<br>47.37	60.61<br>59.92	59	185	167	201	**177**	180	**173**	162	207	184	**177**	**211**	?	Intron (GDF5)		
CX-4344	F=GCCTGATTGATATATGMCTGS<br>R=AAAGAGGTCSYGYTACTTCCA	21<br>21	2<br>3	-<br>-	47.62<br>47.62	59.55<br>59.98	59	212	212	216	210	202	196	204	204	226	241	?	214	5'UTR (NDP)		

F: forward primer; R: reverse primer; L_{prim}: Primer length; DS: number of secondary (W, S, M, K, R and Y) and tertiary (B, D, H and V) degenerate sites; %GC: G+C content; T_m: melting temperature; T_{init}: initial temperature (see methods); *Hsa*: human, Ptr: chimpanzee; *Mmu*: mouse, *Rno*: rat, *Cfa*: dog, *Fca*: cat, *Bta*: cow, *Sar*: shrew, *Eeu*: hedgehog, *Ete*: tenrec, *Meu*: tammar wallaby, *Oan*: platypus; Genotype: indicates if the locus has been genotyped; Seq: indicates if the locus has been sequenced. Expected lengths were calculated based on the 17-WA, or directly from trace files using MegaBLAST (http://www.ncbi.nlm.nih.gov/BLAST/mmtrace.shtml) when applicable, i.e. when the sequence is absent in the 17-WA and for tammar wallaby, which is not included in the 17-WA.
