# Supplementary material for: Design and Implementation of Degenerate Microsatellite Primers for the Mammalian Clade
Source: PLoS One. 2011 Dec 27;6(12):e29582. doi: 10.1371/journal.pone.0029582 (PMC3246486; doi:10.1371/journal.pone.0029582)
Supplement: Information S4 — Distribution of human microsatellites conserved in nine non-primate species. The human data (in bold) correspond to the total number of human microsatellites found to be conserved in at least one species. Species-specific subsets correspond to the number of human microsatellites that are conserved in at least each one of those species. Numbers in brackets indicate numbers for the whole genome (excluding Y chromosome). (PDF) [file pone.0029582.s004.pdf]

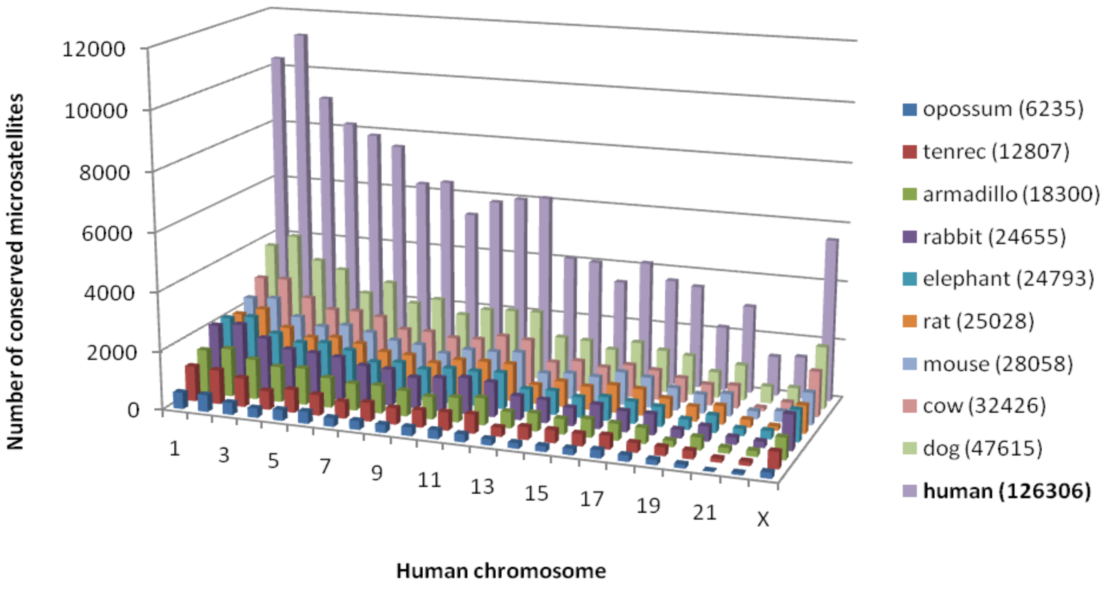

Supporting Information 4: Distribution of human microsatellites conserved in nine non-primate species. The human data (in bold) correspond to the total number of human microsatellites found to be conserved in at least one species. Species-specific subsets correspond to the number of human microsatellites that are conserved in at least each one of those species. Numbers in brackets indicate numbers for the whole genome (excluding Y chromosome).
