# Supplementary material for: Design and Implementation of Degenerate Microsatellite Primers for the Mammalian Clade
Source: PLoS One. 2011 Dec 27;6(12):e29582. doi: 10.1371/journal.pone.0029582 (PMC3246486; doi:10.1371/journal.pone.0029582)
Supplement: Information S5 — Electrophoresis of PCR products for C2-1218 in 17 mammals. Bta: cow, Oar: sheep, Cfa: dog, Mmu: mouse, Fca: cat, Meu: tammar wallaby, Oan: platypus, Gme: pilot whale, Dma: quoll, Rno: rat, Ddu: dugong, Sar: shrew, Eeu: hedgehog, Tac: echidna, Hsa: human, Tad: dolphin, Ete: tenrec. (negative control: water). (PDF) [file pone.0029582.s005.pdf]

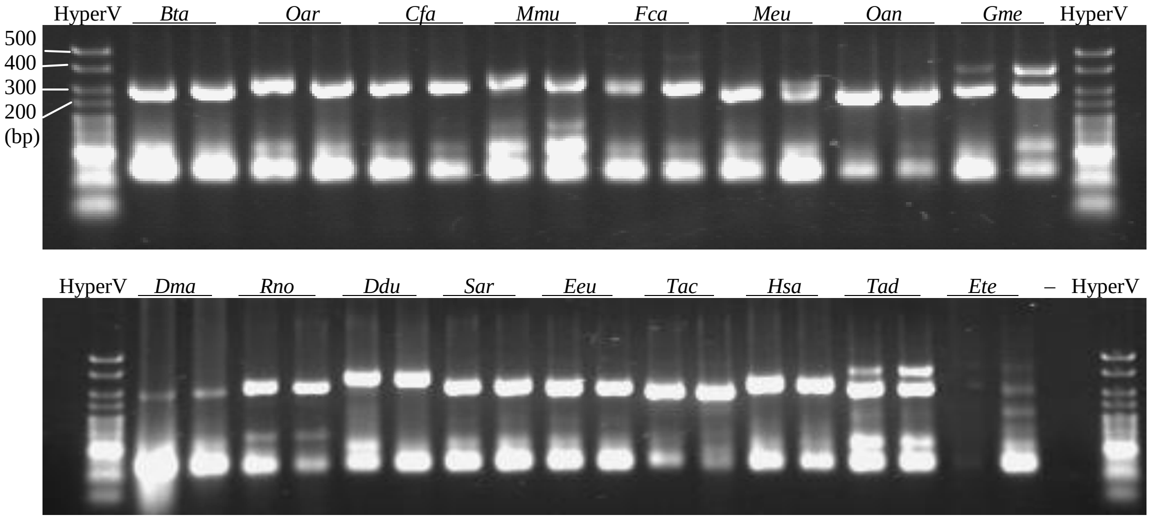

Supporting Information 5: Electrophoresis of PCR products for C2-1218 in 17 mammals. *Bta*: cow, *Oar*: sheep, *Cfa*: dog, *Mmu*: mouse, *Fca*: cat, *Meu*: tammar wallaby, *Oan*: platypus, *Gme*: pilot whale, *Dma*: quoll, *Rno*: rat, *Ddu*: dugong, *Sar*: shrew, *Eeu*: hedgehog, *Tac*: echidna, *Hsa*: human, *Tad*: dolphin, *Ete*: tenrec. (negative control: water).
