# Supplementary material for: Design and Implementation of Degenerate Microsatellite Primers for the Mammalian Clade
Source: PLoS One. 2011 Dec 27;6(12):e29582. doi: 10.1371/journal.pone.0029582 (PMC3246486; doi:10.1371/journal.pone.0029582)
Supplement: Information S6 — Polymorphic Information Content (PIC) at nine cross-species microsatellite loci based on at most 20 individuals per species. (PDF) [file pone.0029582.s006.pdf]

Supporting Information 6: Polymorphic Information Content (PIC) at nine cross-species microsatellite loci based on at most 20 individuals per species.

	C2-1218*	C2-6868*	C2-1915*	C4-1514*	C6-1112	C9-1918	C14-9692	C15-3531	C17-4243*
**Human**	0.78	0	0.56	0.22	0.17	0.38	0.14	0.22	0
**Chimpanzee**	n/a	n/a	n/a	n/a	n/a	n/a	n/a	n/a	n/a
**Mouse**	0.71	0.90	0.87	0.46	0.40	0	0.45	0.11	0.53
**Rat**	0.29	0	0.28	0	0.47	n/a	n/a	0	0
**Dog**	0.79	0.46	0.73	0.11	n/a	n/a	0	n/a	0
**Cat**	0.78	n/a	0.65	n/a	n/a	n/a	n/a	n/a	0
**Cow**	0.05	0	0.20	0	0	0.37	0	0.05	0
**Sheep**	0.78	0.33	0.57	0	0	0.09	0.51	n/a	0
**Dolphin**	0.53	n/a	0.76	0.11	0.21	0.48	0.23	0	0.31
**Pilot Whale**	0	0	0.73	0	0	0.62	0	0	0
**Hedgehog**	0.62	0.10	0.46	0.36	0.25	0	0.25	0.76	0
**Shrew**	0.84	0.36	0	0	n/a	n/a	n/a	n/a	0.23
**Dugong**	0.50	0.05	0	0	0	0	n/a	0	0.35
**Tenrec**	n/a	n/a	n/a	0	n/a	n/a	n/a	n/a	0.45
**Tammar wallaby**	0.77	n/a	0.06	0	0	n/a	n/a	0.90	0.60
**Quoll**	0.10	0.72		0	0	n/a	0	n/a	0
**Platypus**	0.12	0.64	0.32		0	n/a	0	n/a	0
**Echidna**	0.51	0.58		0	0	0	0.69	0.08	0

* Sequenced loci; n/a : data not available
