# Supplementary material for: Design and Implementation of Degenerate Microsatellite Primers for the Mammalian Clade
Source: PLoS One. 2011 Dec 27;6(12):e29582. doi: 10.1371/journal.pone.0029582 (PMC3246486; doi:10.1371/journal.pone.0029582)
Supplement: Information S7 — UCSC 28-way alignment of five cross-species microsatellite locus showing species of interest (in order: C2-1218; C2-6868; C2-1915; C4-1514; C17-4243). (A) Flanking sequences. Underscores represent the microsatellite sequence; positions are counted upstream and downstream from the microsatellite. Boxes indicate primer sites, dashes gaps and dots bases identical to human. (B) Microsatellite sequence. Array length is shown in brackets. UCSC assemblies: Human (hg18), chimp (panTro2), mouse (mm8), rat (rn4), cow (bosTau3), dog (canFam2), cat (felCat3), shrew (sorAra1), hedgehog (eriEur1), armadillo (dasNov1), elephant (loxAfr1), tenrec (echTel1), opossum (monDom4), platypus (ornAna1). (PDF) [file pone.0029582.s007.pdf]

Supporting Information 7: UCSC 28-way alignment of five cross-species microsatellite locus showing species of interest (in order: C2-1218; C2-6868; C2-1915; C4-1514; C17-4243). (A) Flanking sequences. Underscores represent the microsatellite sequence; positions are counted upstream and downstream from the microsatellite. Boxes indicate primer sites, dashes gaps and dots bases identical to human. (B) Microsatellite sequence. Array length is shown in brackets. UCSC assemblies: Human (hg18), chimp (panTro2), mouse (mm8), rat (rn4), cow (bosTau3), dog (canFam2), cat (felCat3), shrew (sorAra1), hedgehog (eriEur1), armadillo (dasNov1), elephant (loxAfr1), tenrec (echTel1), opossum (monDom4), platypus (ornAna1).

A

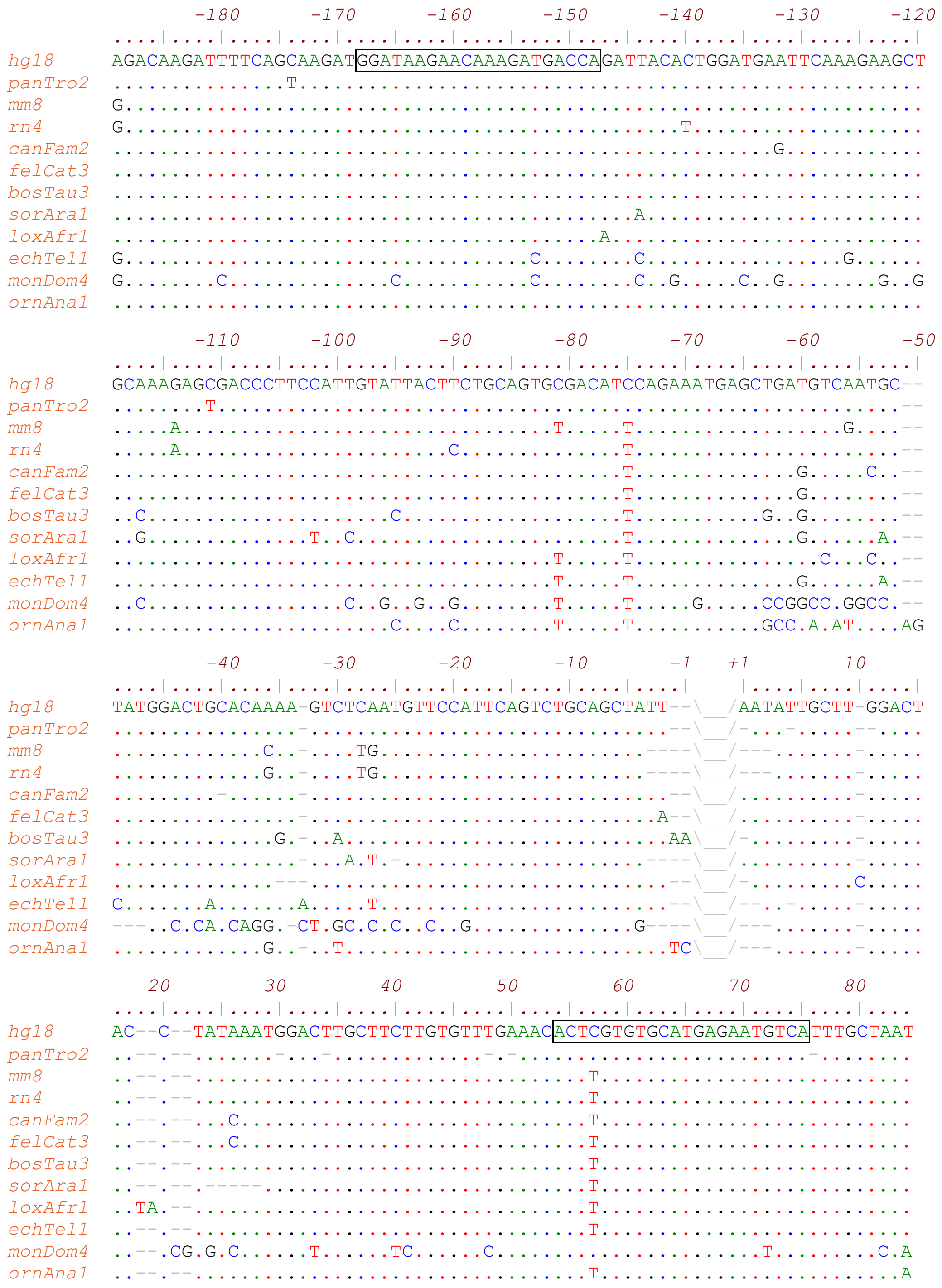

B

hg18	(46)	\ $(CA)_{23}$ /
panTro2	(49)	\ $(CA)_{24}$/
mm8	(65)	\ $(CA)_{15}(CCACACC)_{4}C(CA)_{3}$/
rn4	(53)	\ $(CA)_{20}CTA(CA)_{6}$ /
canFam2	(38)	\ $(CA)_{4}TA(CA)_{10}CG(CA)_{3}$/
felCat3	(38)	\ $(CA)_{19}$/
bosTau3	(31)	\ $(CA)_{15}G$/
sorAra1	(89)	\ $(AC)_{19}AT(AC)_{5}GC(AC)_{5}ATGC(AC)_{2}A(AC)_{4}AT(AC)_{2}(AT)_{2}$/
loxAfr1	(34)	\ $(CA)_{4}TA(CA)_{4}TA(CA)_{7}$/
echTel1	(33)	\ $(CA)_{16}C$/
monDom4	(39)	\ $(CA)_{2}CTCG(CCCACA)_{2}(CTCACA)_{2}(CA)_{2}CCA$/
ornAna1	(12)	\ $(CA)_{4}(A)_{4}$/

C2-1218

A

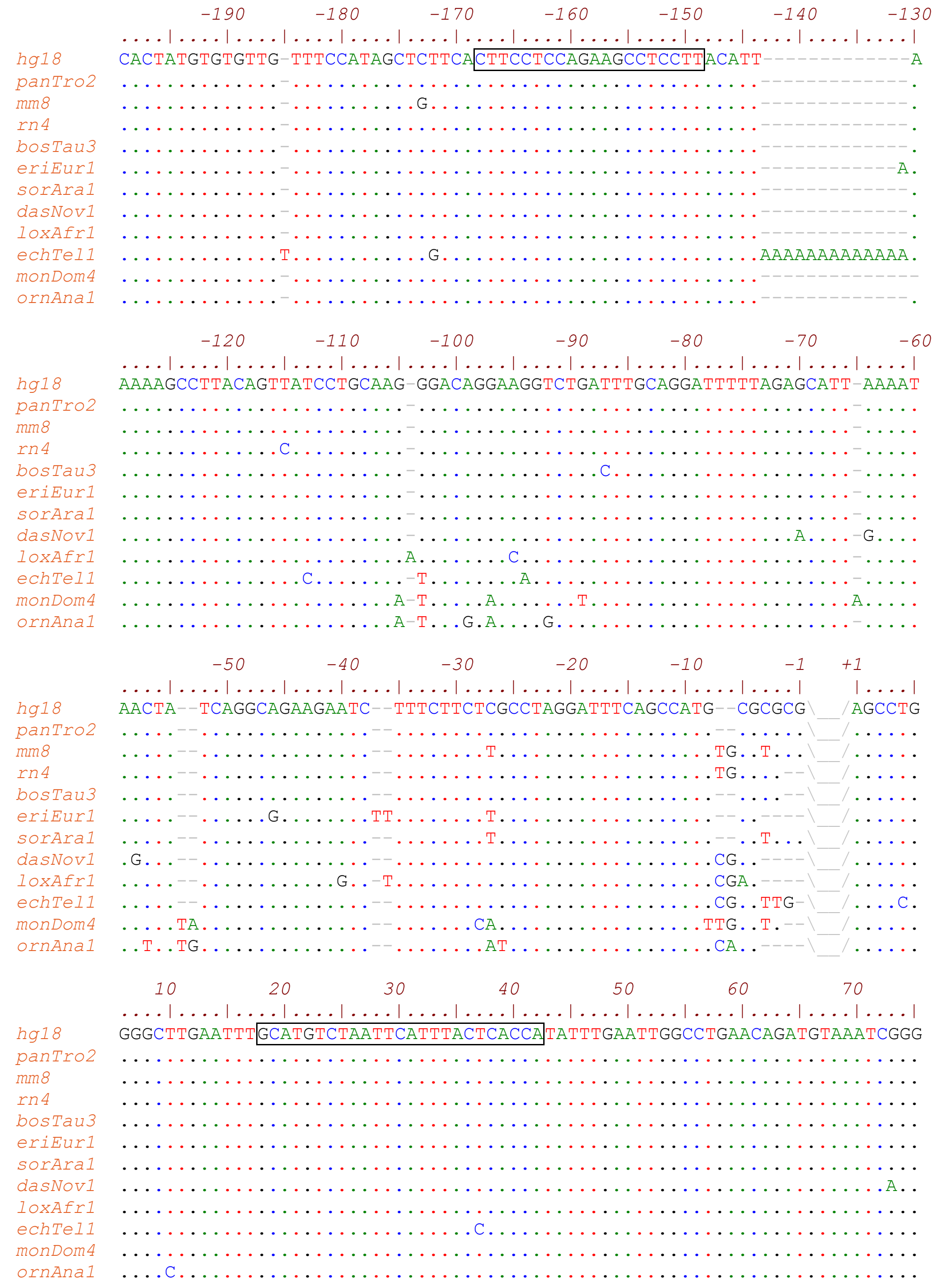

B

hg18	(42)	\(CT)$_5$TT(CT)$_4$(T)$_4$C(CT)$_4$CC(CT)$_2$TTCT/
panTro2	(42)	\(CT)$_5$TT(CT)$_4$(T)$_4$C(CT)$_4$CC(CT)$_2$TTCT/
mm8	(80)	\(CT)$_{29}$T(CTC)$_2$C(CT)$_2$CC(CT)$_2$TTCT/
rn4	(50)	\(CT)$_7$CC(CT)$_6$(TC)$_2$(CT)$_4$CC(CT)$_4$/
bosTau3	(42)	\(CT)$_5$TT(CT)$_5$(T)$_4$(CT)$_4$CC(CT)$_4$/
eriEur1	(40)	\(CT)$_5$AT(CT)$_4$(T)$_3$C(CT)$_5$CC(CT)$_4$/
sorAra1	(64)	\(CT)$_3$TT(CT)$_5$TT(CT)$_4$CCTT(CT)$_5$TCTT(CT)$_4$CC(CT)$_4$/
dasNov1	(42)	\(CT)$_5$TT(CT)$_4$(T)$_3$C(CT)$_4$CC(CT)$_4$/
loxAfr1	(42)	\(CT)$_5$TT(CT)$_5$TTC(CT)$_4$CC(CT)$_4$/
echTel1	(71)	\ (CT)$_{22}$T(CT)$_3$TC(CT)$_4$CC(CT)$_2$TTCT/
monDom4	(42)	\(CT)$_{11}$TT(CT)$_4$CCTT(CT)$_3$/
ornAna1	(166)	\(CT)$_3$TGCTCATCTTTCTGGCCTGT(CT)$_2$(C)$_5$(CT)$_2$GTC(T)$_4$G(CT)$_2$(CCCT)$_2$(CT)$_4$\\ \\CC(CT)$_5$(C)$_8$TTACA(CT)$_6$AC(CT)$_3$CCT(C)$_4$ATGACTGTTCCCCTCCCATTTCT\\ \\(C)$_9$AT(CT)$_4$TCCT/

C2-6868

A

```
              -140      -130      -120      -110      -100       -90       -80
         ....|....|....|....|....|....|....|....|....|....|....|....|....|....|
hg18     AATAGTACAGACATGCCATCTTGTAACACTATATGCCTGTCAGGAGGGACAGGGCCTGGTGG----AGCA
panTro2  ..............................................................----....
mm8      ...................................................-...AG...T.----....
rn4      ................................................G..-...AG...T.----....
bosTau3  ............................................................TA----....
canFam2  ............................................................CA----....
felCat3  ----------------------------------------------------------------------
eriEur1  .....................................................C......T.----....
sorAra1  ...............................C.C........................C.T.----....
dasNov1  .....C..............................AC............CA........T.----....
monDom4  ..........G................................A..A.C..A...T....A.----....
ornAna1  ..............C........C........C..........A....C.......G.C.T.CCAC....

               -70       -60       -50       -40       -30       -20       -10
         ....|....|....|....|....|....|....|....|....|....|....|....|....|....|
hg18     TCAGCTATATAAGGCTGGTAACTAGGGGAA-GCCCAACAAACAGCCACAAACACCTAAGTTATTTTACTC
panTro2  ..............................-.......................................
mm8      ...A.......................A..G...............................C.......
rn4      ...A......................A...G...............................C.......
bosTau3  ...A.......................A..-.........................C.............
canFam2  ...A.......................A..-.......................................
felCat3  ----------------------------------------------------------------------
eriEur1  ...A.......................A..-.......................................
sorAra1  ...A..................C.......-.........................C.............
dasNov1  ..............T.......C.......-...............C.......................
monDom4  .A..............T.............-..............G........................
ornAna1  GG..............T.............-.....G...................T..........T..

               -1    +1        10        20        30        40        50
         ....|....|    |....|....|....|....|....|....|....|....|....|....|....|
hg18     AGCTACTACC\__/tTTTCATCTTGGCTTTCTGTGGCAGAATGCAAATGGAGCCTGCCGGCGGTGAAAGGG
panTro2  ..........\__/.........................................................
mm8      .A.C......\__/...................................................-....A
rn4      .A.C......\__/.........................................................
bosTau3  ..........\__/.........................................................
canFam2  ..........\__/....................A....................................
felCat3  ----------\__/------------------------------..............C.T..........
eriEur1  .......C..\__/.........................................................
sorAra1  .........A\__/.........................................................
dasNov1  ...C......\__/.........................................................
monDom4  .C.CT.C...\__/.........G.........................T.....................
ornAna1  ...CT.C...\__/...................................T.....A...............

              60        70        80        90       100       110       120
         ....|....|....|....|....|....|....|....|....|....|....|....|....|....|
hg18     CTGAATTGTGATTAAGAAGAAGAAGAGCTCCTTTCTTTGTTCTCCCCTCAGGGGATGTTTACTGGGAGAG
panTro2  ......................................................................
mm8      .............................-.............T..........................
rn4      .............................-........................................
bosTau3  ......................................................................
canFam2  ......................................................................
felCat3  ......................................................................
eriEur1  ......................................................................
sorAra1  ......................................................................
dasNov1  ......................................................................
monDom4  ......................................................................
ornAna1  ...........................T...............G.........A................
```

B

hg18 (51) \CTGTGC(CT)$_{15}$TT(TC)$_{5}$TTC/

panTro2 (37) \CTGTGC(CT)$_{14}$TTCT/

mm8 (75) \(CT)$_{2}$GAGC(CT)$_{2}$CC(CT)$_{14}$GT(CT)$_{2}$GT(CT)$_{12}$C/

rn4 (53) \(CT)$_{2}$GC(CT)$_{23}$C/

bosTau3 (50) \(CT)$_{2}$GC(CT)$_{2}$T(CT)$_{7}$(C)$_{4}$(CT)$_{9}$TTC/

canFam2 (61) \(CT)$_{3}$(CCT)$_{2}$(CTCTCCT)$_{2}$(CT)$_{16}$TTC/

felCat3 –

eriEur1 (50) \(CT)$_{2}$GCCTGCTT(CT)$_{15}$TTCTTTC/

sorAra1 (105) \CAT(TC)$_{2}$TG(TC)$_{2}$TG(CCTCT)$_{2}$GC(CTCTGT)$_{2}$(CT)$_{4}$(GTCTCT)$_{2}$(CTGTCT)$_{2}$\\
\\(CT)$_{7}$(C)$_{3}$(T)$_{3}$(C)$_{3}$(TC)$_{2}$CC(TC)$_{3}$TTTC/

dasNov1 (67) \(CT)$_{2}$GCCTT(CT)$_{9}$GT(CT)$_{3}$GT(CT)$_{5}$TT(CT)$_{8}$TTC/

monDom4 (153) \(CT)$_{2}$GCT(C)$_{6}$ACC(T)$_{3}$(CT)$_{19}$CA(CT)$_{44}$A(T)$_{4}$/

ornAna1 (100) \ CTGTTC(T)$_{6}$C(A)$_{5}$(T)$_{5}$ATC(CCT)$_{7}$(CTT)$_{2}$T(CT)$_{2}$T(CT)$_{2}$CC(CT)$_{4}$TT(CT)$_{5}$(T)3A(T)$_{7}$CCTC /

C2-1915

A

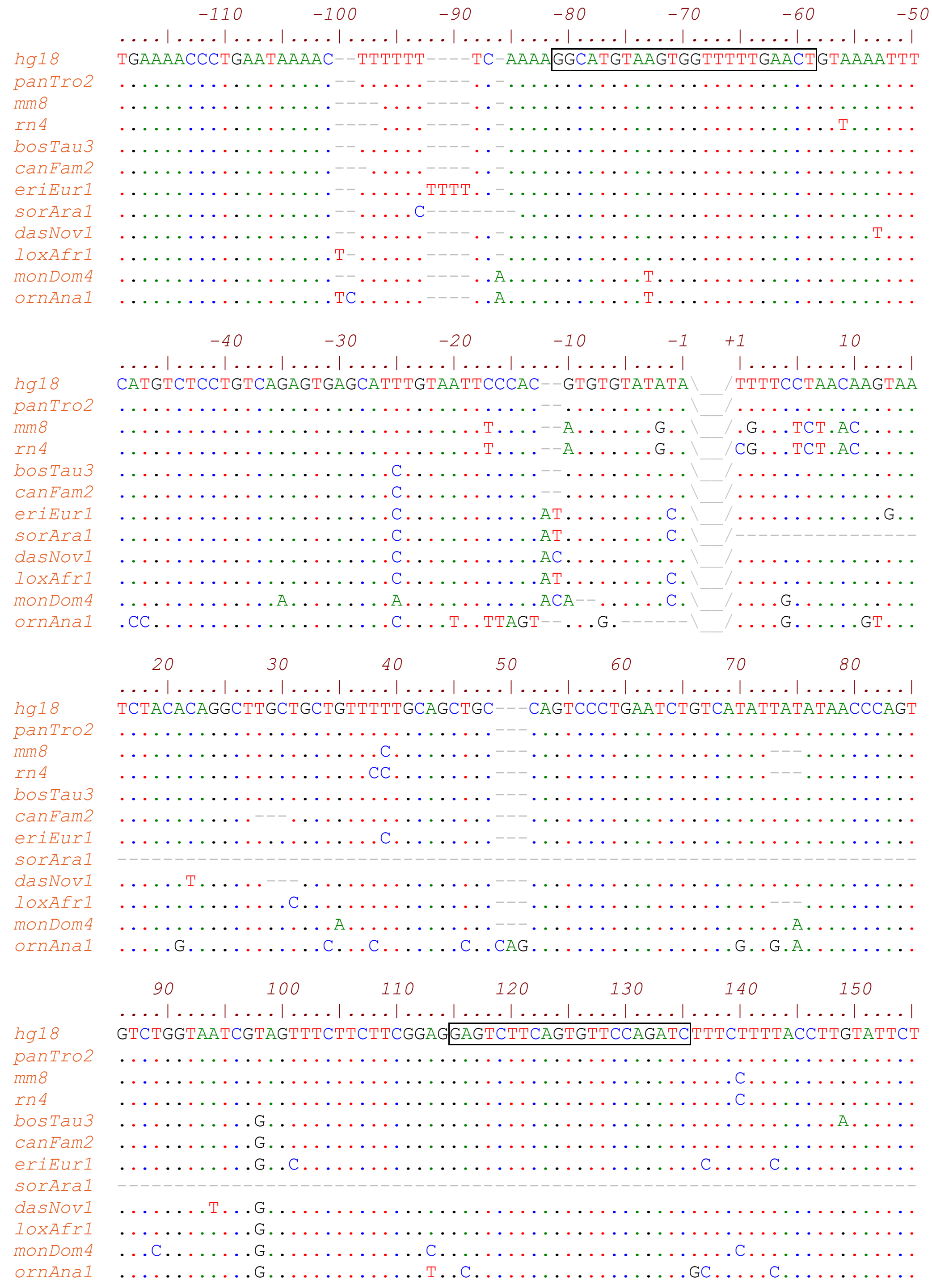

B

hg18 (70) \GA(CA)$_{3}$AATA(CA)$_{2}$CC(TG)$_{2}$(CA)$_{2}$TA(CA)$_{4}$CGC(A)$_{3}$(CA)$_{9}$CT(CA)$_{2}$AACA/

panTro2 (71) \GA(CA)$_{3}$AATA(CA)$_{2}$CC(TG)$_{2}$(CA)$_{2}$TA(CA)$_{4}$GC(A)$_{3}$(CA)$_{9}$C(CA)$_{3}$AACA/

mm8 (104) \GATA(CA)$_{5}$AAGACT(CA)$_{2}$TA(C)$_{4}$TGTA(CA)$_{2}$TACATTCCT(GCAC)$_{2}$A(CG)$_{3}$(CA)$_{4}$(CT)$_{2}$(CA)$_{10}$\\
\\GTCTCG(CA)$_{2}$/

rn4 (66) \GATA(CA)$_{6}$AAGACT(CA)$_{3}$(C)$_{3}$TTGTA(CA)$_{3}$CC(CA)$_{5}$CC(CA)$_{5}$/

bosTau3 (66) \GA(CA)$_{3}$(A)$_{4}$(CA)$_{2}$CC(TG)$_{2}$(CA)$_{2}$TA(CA)$_{2}$AA(CA)$_{3}$TACACGCTCACG(CA)$_{2}$TACA(C)$_{3}$G/

canFam2 (82) \GA(CA)$_{3}$AA(CA)$_{3}$CC(TG)$_{2}$(CA)$_{2}$TA(CA)$_{6}$CGCCAACG(CA)$_{2}$TA(CA)$_{3}$CG(CA)$_{2}$TA(CA)$_{5}$AACA/

eriEur1 (107) \(CA)$_{4}$(A)$_{3}$TA(CA)$_{3}$CC(T)$_{3}$(GC)$_{2}$AC(GC)$_{2}$GT(GC)$_{3}$A(CA)$_{2}$TA(CA)$_{12}$TATGC(A)$_{3}$(CA)$_{7}$TA(CA)$_{3}$AATA/

sorAra1 -

dasNov1 (48) \GACACGC(A)$_{3}$(CA)$_{3}$CC(TG)$_{2}$(CA)$_{2}$TA(CA)$_{4}$CG(CA)$_{3}$TACA/

loxAfr1 (64) \GA(CA)$_{3}$AATA(CA)$_{2}$CC(TG)$_{2}$(CA)$_{2}$TA(CA)$_{4}$CGC(A)$_{3}$(CA)$_{6}$(CG)$_{2}$C(A)$_{3}$CA/

monDom4 (75) \GA(CATACA)$_{2}$T(C)$_{3}$AGTGCG(CA)$_{2}$CG(CA)$_{5}$CG(CA)$_{2}$CT(CA)$_{3}$(TA)$_{2}$(CAAA)$_{2}$(CA)$_{3}$TCA/

ornAna1 (16) \(C)$_{10}$(A)$_{4}$CA/

C4-1514

A

```
                -150      -140      -130      -120      -110      -100       -90
          ....|....|....|....|....|....|....|....|....|....|....|....|....|....|
hg18      ATGGCTATTTGTCACATTTTACGACAATAACATTAATAACAAACAATAAATTTACATGGACATATAAGAC
panTro2   ......................................................................
mm8       ....................................................................G.
rn4       ...C.........................G...............................G..C...G.
bosTau3   ......................................................................
canFam2   ......................................................................
eriEur1   ----------------------------------------------------------------------
sorAra1   ................................................................G.....
loxAfr1   ......................................................................
echTel1   ......................................................................
monDom4   ......................................................................
ornAna1   GC......C.A.............................................C.....C..-....

                 -80       -70       -60       -50       -40       -30       -20
          ....|....|....|....|....|....|....|....|....|....|....|....|....|....|
hg18      GCGGTAGGTAGT-GAGAAATCCCTTCTACTTACAATACCCCAGCCCGCGGTG-TGGCTCCGGCTGCGGGG
panTro2   ............-.......................................-.................
mm8       ............-.....................G.................-...T...A....T....
rn4       ............-.....................G.................-...T...A.........
bosTau3   ............-.......................................-.................
canFam2   ............-....................................A..-.................
eriEur1   ------------------------------------------------------------------....
sorAra1   ............-......C................................-.................
loxAfr1   ............-....................................A..-.............-...
echTel1   ............-.......................................-.......A..G..A...
monDom4   A...........-........................A........A..T..-...........CT....
ornAna1   A..........CG.G.........CT...........A........A.....-..CTGTA....CTC.C.

                 -10        -1    +1        10        20        30        40
          ....|....|....|....|    |....|....|....|....|....|....|....|....|....|
hg18      CTGTTTTATTGCTGtctccc\__/-GGGGTACAGGCTTG-TTTTTCAAA-GGACAGTTGAATTGCACGTC
panTro2   ....................\__/-..............-.........-....................
mm8       A.C.....C..A..C.....\__/-A.............-.........-..............G.....
rn4       ..C.....CA.A..C.....\__/-A.............T.........-..............G.....
bosTau3   ..............C.....\__/-..............-.........-....................
canFam2   ....................\__/-..............-.........-....................
eriEur1   ..............c.....\__/A.....CA.....C.-.....T...G.......C............
sorAra1   ..............c.....\__/-..............-.........-...............T....
loxAfr1   .G............C.....\__/-..............-.........-....................
echTel1   .A........A...C.....\__/-..............-.........-....................
monDom4   ..C...........C.....\__/-...A..T.......-.....T...-....................
ornAna1   .C........A.C.C.....\__/-......T.......-.....T...-....................

             50        60        70        80        90       100       110
          ....|....|....|....|....|....|....|....|....|....|....|....|....|....|
hg18      AGAAACAGGGAGA-CCGAGCCTGC--GATTTTCCTGACGAATACATATCTATTCTGCAACCTCGGCATTA
panTro2   .............-..........--............................................
mm8       .............-..........--....................................A.......
rn4       .............-..........--............................................
bosTau3   .............-.........T--............................................
canFam2   .............-..........--............................................
eriEur1   ..C..........C..........CGAT..........................................
sorAra1   .............-..........--............................................
loxAfr1   .............-..A.......--A...........................................
echTel1   .............-..A.......--.....................G......................
monDom4   .............-..AT.T....--.....................................T......
ornAna1   .............-..AT.T....--.....................................T......
```

B

hg18	(55) \(TTTC)$_{2}$(TC)$_{7}$CC(TC)$_{5}$(C)$_{5}$(TC)$_{2}$(T)$_{8}$(TC)$_{2}$/
panTro2	(57) \(TTTC)$_{2}$(TC)$_{7}$CC(TC)$_{5}$(C)$_{4}$(TC)$_{3}$(T)$_{9}$(TC)$_{2}$/
mm8	(55) \(TTTC)$_{2}$(TC)$_{15}$CC(TC)$_{3}$(T)$_{5}$(TC)$_{2}$/
rn4	(60) \TT(TC)$_{19}$CC(TC)$_{2}$(T)$_{10}$CCTC
bosTau3	(50) \(TC)$_{2}$(T)$_{4}$(TC)$_{6}$CC(TC)$_{4}$(C)$_{4}$(TC)$_{2}$(T)$_{8}$(TC)$_{2}$/
canFam2	(51) \(TC)$_{2}$(T)$_{4}$(TC)$_{7}$CC(TC)$_{3}$(C)$_{4}$(TC)$_{3}$(T)$_{7}$(TC)$_{2}$/
eriEur1	(52) \(TTC)$_{2}$TA(TCC)$_{2}$T(TCC)$_{2}$CCTT(TCT)$_{2}$(C)$_{5}$(TCT)$_{2}$(T)$_{5}$(TC)$_{2}$/
sorAra1	(58) \(TC)$_{2}$TT(TC)$_{9}$CC(TC)$_{6}$(C)$_{4}$(TC)$_{2}$(T)$_{8}$(TC)$_{2}$/
loxAfr1	(54) \(TC)$_{2}$(T)$_{4}$(TC)$_{5}$CC(TC)$_{5}$(C)$_{4}$(TC)$_{3}$(T)$_{10}$(TC)$_{2}$/
echTel1	(68) \TGTCTT(TC)$_{8}$CC(TC)$_{3}$TT(TC)$_{2}$(C)$_{4}$(TC)$_{3}$(C)$_{3}$(TC)$_{6}$(T)$_{5}$(TC)$_{2}$/
monDom4	(55) \TCTT(TC)$_{9}$TT(TC)$_{5}$(C)$_{4}$(TC)$_{4}$(T)$_{5}$(TC)$_{2}$/
ornAna1	(42) \(TC)$_{2}$(T)$_{4}$(TC)$_{4}$CCTTGC(TC)$_{2}$CC(TC)$_{3}$(T)$_{5}$TCTT/

C17-4243
