# Supplementary material for: Design and Implementation of Degenerate Microsatellite Primers for the Mammalian Clade
Source: PLoS One. 2011 Dec 27;6(12):e29582. doi: 10.1371/journal.pone.0029582 (PMC3246486; doi:10.1371/journal.pone.0029582)
Supplement: Information S8 — Allele length, and microsatellite length and sequence variation in at most four individuals for five cross-species microsatellite loci. Lallele: allele length (bp); Lmicro: microsatellite length (bp); an individual (Indiv 1) 1 is used as a reference to measure length variation (+/−). (PDF) [file pone.0029582.s008.pdf]

Supporting Information 8: Allele length, and microsatellite length and sequence variation in at most four individuals for five cross-species microsatellite loci. L_{allele}: allele length (bp); L_{micro}: microsatellite length (bp); an individual (Indiv 1) is used as a reference to measure length variation (+/-).

C2-1218

Species	Indiv	L_{allele}	L_{micro}	Sequence
Human	1	280	48	$(CA)_{24}$
	2,4	+1	0	$(CA)_{24}$
	3	-2	-2	$\mathbf{(CA)_{23}}$
Chimp	1,3	*n/a*	50	$(CA)_{25}$
	2		-8	$\mathbf{(CA)_{19}}$
	4		+4	$\mathbf{(CA)_{27}}$
Mouse	1	291	64	$(CA)_{18}(CCACACC)_{3}C(CA)_{3}$
	2	+5	+5	$\mathbf{(CA)_{17}(CCACACC)_{4}}C(CA)_{3}$
	3	+9	+9	$\mathbf{(CA)_{19}(CCACACC)_{4}}C(CA)_{3}$
	4	+7	+7	$(CA)_{18}\mathbf{(CCACACC)_{4}}C(CA)_{3}$
Rat	1,2,3,4	276	49	$(CA)_{17}CTA(CA)_{6}$
Dog	1	274	44	$(CA)_{4}TA(CA)_{13}CG(CA)_{3}$
	2,4	-6	-6	$(CA)_{4}TA\mathbf{(CA)_{10}}CG(CA)_{3}$
	3	-4	-4	$(CA)_{4}TA\mathbf{(CA)_{11}}CG(CA)_{3}$
Cat	1	265	38	$(CA)_{19}$
	2,3,4	+6	+2	$\mathbf{(CA)_{20}}$
Cow	1	269	28	$(CA)_{12}(GA)_{2}$
	3,4	0	0	$\mathbf{(CA)_{13}GA}$
Sheep	1	276	43	$(CA)_{11}CG(CA)_{4}(C)_{3}(CA)_{4}$
	2,4	-6	-6	$\mathbf{(CA)_{13}}(C)_{3}(CA)_{4}$
	3	+2	+2	$\mathbf{(CA)_{12}}CG(CA)_{4}(C)_{3}(CA)_{4}$
Dolphin	1	264	32	$(CA)_{16}$
	2	+4	+4	$\mathbf{(CA)_{18}}$
Pilot Whale	1,2,3,4	265	34	$(CA)_{8}TA(CA)_{8}$
Hedgehog	2	260	40	$(CA)_{20}$
	3	0	+4	$\mathbf{(CA)_{22}}$
	4	+8	-2	$\mathbf{(CA)_{19}}$
Dugong	1	270	38	$(CA)_{4}CG(CA)_{14}$
	2,4	+1	+2	$(CA)_{4}CG\mathbf{(CA)_{15}}$
	3	+2	0	$(CA)_{4}CG\mathbf{(CA)_{15}}$
T. wallaby	1,3	251	41	$(CA)_{13}CG(CA)_{5}ACA$
Platypus	2,3,4	245	12	$(CA)_{4}(A)_{4}$

C2-6868

Species	Indiv	L_{allele}	L_{micro}	Sequence
Human	1,2	228	42	$(CT)_{5}TT(CT)_{4}(T)_{4}C(CT)_{4}CC(CT)_{2}TTCT$
Chimp	1,2,3,4	*n/a*	42	$(CT)_{5}TT(CT)_{4}(T)_{4}C(CT)_{4}CC(CT)_{2}TTCT$
Rat	2,3	236	50	$(CT)_{7}CC(CT)_{6}(TC)_{2}(CT)_{4}CC(CT)_{4}$
Dog	4	262	76	$(CT)_{8}CCTT(CT)_{12}TT(CT)_{3}TTCC(CT)_{5}CC(CT)_{4}$
Cow	1,2,3,4	231	44	$(CT)_{5}TT(CT)_{5}(T)_{4}(CT)_{4}CC(CT)_{4}$
Sheep	2	231	44	$(CT)_{5}TT(CT)_{5}(T)_{4}(CT)_{4}CC(CT)_{4}$
	4	-2	-2	$(CT)_{5}TT\mathbf{(CT)_{4}}(T)_{4}(CT)_{4}CC(CT)_{4}$
Dolphin	3,4	*n/a*	56	$(CT)_{5}GTC(T)_{3}(CT)_{9}(T)_{3}C(CT)_{4}CC(CT)_{4}$
Pilot Whale	3	243	56	$(CT)_{5}GTC(T)_{3}(CT)_{9}(T)_{3}C(CT)_{4}CC(CT)_{4}$
Hedgehog	3,4	230	44	$(CT)_{5}AT(CT)_{4}(T)_{3}C(CT)_{5}CC(CT)_{4}$
Shrew	1,4	256	68	$(CT)_{3}TT(CT)_{7}TT(CT)_{4}CCTT(CT)_{5}TCTT(CT)_{4}CC(CT)_{4}$
	2	-2	0	$(CT)_{3}TT(CT)_{7}TT(CT)_{4}CCTT(CT)_{5}TCTT(CT)_{4}CC(CT)_{4}$
Dugong	1,3,4	225	40	$(CT)_{5}TT(CT)_{3}(T)_{3}C(CT)_{4}TC(CT)_{4}$

C2-1915

Species	Indiv	L_{allele}	L_{micro}	Sequence
Human	1,2,3,4	283	49	CTGTGC(CT)$_{14}$TT(TC)$_{5}$TTC
Chimp	1,2,3	*n/a*	37	CTGTGC(CT)$_{14}$TTC
	4		+2	CTGTGC**(CT)$_{15}$**TTC
Rat	2,3,4	273	57	(CT)$_{2}$GC(CT)$_{25}$C
Cow	1,3,4	169	50	(CT)$_{2}$GC(CT)$_{2}$T(CT)$_{8}$CC(CT)$_{9}$TTC
	2	-2	0	(CT)$_{2}$GC(CT)$_{2}$T(CT)$_{8}$CC(CT)$_{9}$TTC
Sheep	2,4	181	58	(CT)$_{2}$GC(CT)$_{2}$T(CT)$_{12}$CC(CT)$_{9}$TTC
	3	-8	-8	(CT)$_{2}$GC(CT)$_{2}$T**(CT)$_{10}$**CC**(CT)$_{8}$C**
Dolphin	1,3,4	172	47	(CT)$_{22}$TTC
	2	-4	-4	**(CT)$_{20}$**TTC
Hedgehog	1,4	168	49	(CT)$_{2}$GCCTGCTT(CT)$_{15}$TTC(T)3C
	2,3	+2	+2	(CT)$_{2}$GCCTGCTT**(CT)$_{16}$**TTC(T)3C
Shrew	1	280	99	CAT(TC)$_{2}$TG(TC)$_{2}$TG(CCTCT)$_{2}$GC(CTCTGT)$_{2}$(CT)$_{4}$(GTCTCT)$_{2}$(CTGTCT)$_{2}$\\<br>\\(CT)$_{6}$(C)$_{3}$(T)$_{3}$(CCCTCT)$_{1}$(CT)$_{2}$TTC
	3	0	+2	CAT(TC)$_{2}$TG(TC)$_{2}$TG(CCTCT)$_{2}$GC(CTCTGT)$_{2}$(CT)$_{4}$(GTCTCT)$_{2}$(CTGTCT)$_{2}$\\<br>**(CT)$_{7}$**(C)$_{3}$(T)$_{3}$(CCCTCT)$_{1}$(CT)$_{2}$TTC
Dugong	1,2,3,4	274		(CT)$_{2}$GC(CT)$_{8}$GC(CT)$_{3}$TT(CT)$_{3}$CC(CT)$_{7}$TTC
Platypus	1,4	223	103	CTGTTC(T)$_{6}$C(A)$_{5}$(T)$_{5}$ATC(CCT)$_{8}$(CTT)$_{2}$T(CT)$_{2}$T(CT)$_{2}$CC(CT)$_{4}$TT(CT)$_{5}$(T)3A(T)$_{7}$CCTC
	2	-10	-9	CTGTTC(T)$_{6}$C(A)$_{5}$(T)$_{5}$ATC**(CCT)$_{5}$**(CTT)$_{2}$T(CT)$_{2}$T(CT)$_{2}$CC(CT)$_{4}$TT(CT)$_{5}$(T)3A(T)$_{7}$CCTC
	3	-3	-3	CTGTTC(T)$_{6}$C(A)$_{5}$(T)$_{5}$ATC**(CCT)$_{7}$**(CTT)$_{2}$T(CT)$_{2}$T(CT)$_{2}$CC(CT)$_{4}$TT(CT)$_{5}$(T)3A(T)$_{7}$CCTC

C4-1514

Species	Indiv	L_{allele}	L_{micro}	Sequence
Human	1,2,3,4	283	72	GA(CA)$_{3}$AATA(CA)$_{2}$CC(TG)$_{2}$(CA)$_{2}$TA(CA)$_{4}$CGC(A)$_{3}$(CA)$_{8}$CT(CA)$_{2}$AACA
Chimp	1,2	*n/a*	70	GA(CA)$_{3}$AATA(CA)$_{2}$CC(TG)$_{2}$(CA)$_{2}$TA(CA)$_{4}$CGC(A)$_{3}$(CA)$_{12}$AACA
	3		+2	GA(CA)$_{3}$AATA(CA)$_{2}$CC(TG)$_{2}$(CA)$_{2}$TA(CA)$_{4}$CGC(A)$_{3}$**(CA)$_{11}$**AACA
	4		-1	GA(CA)$_{3}$AATA(CA)$_{2}$CC(TG)$_{2}$(CA)$_{2}$TA(CA)$_{4}$CGC(A)$_{3}$**(CA)$_{9}$C(AACA)$_{2}$**
Mouse	1,4	315	104	GATA(CA)$_{5}$AAGACT(CA)$_{2}$TA(C)$_{4}$TGTA(CA)$_{2}$TACATTCCT(GCAC)$_{2}$A(CG)$_{3}$(CA)$_{4}$//<br>//(CT)$_{2}$(CA)$_{10}$GTCTCG(CA)$_{2}$
	2,3	+2	+2	GATA(CA)$_{5}$AAGACT(CA)$_{2}$TA(C)$_{4}$TGTA(CA)$_{2}$TACATTCCT(GCAC)$_{2}$A**(CG)$_{4}$(CA)$_{3}$**//<br>//**CT(CA)$_{12}$**GTCTCG(CA)$_{2}$
Rat	1,2,3	274	66	GATA(CA)$_{6}$AAGACT(CA)$_{3}$(C)$_{3}$TTGTA(CA)$_{3}$CC(CA)$_{5}$CC(CA)$_{5}$
	4	0	0	GATA(CA)$_{6}$AAGACT**(CA)$_{4}$C**TTGTA(CA)$_{3}$CC(CA)$_{5}$CC(CA)$_{5}$
Dog	1,2,3,4	297	86	GA(CA)$_{3}$AA(CA)$_{3}$CC(TG)$_{2}$(CA)$_{2}$TA(CA)$_{8}$CGCCAACG(CA)$_{2}$TA(CA)$_{3}$CG(CA)$_{2}$TA//<br>//(CA)$_{5}$AACA
Cow	1,2,3,4	280	70	GA(CA)$_{3}$(A)$_{4}$(CA)$_{2}$CC(TG)$_{2}$(CA)$_{2}$TA(CA)$_{2}$CGC(A)$_{3}$(CA)$_{3}$TACACGCTCACG(CA)$_{2}$TA//<br>//CA(C)$_{3}$G
Sheep	1,2,3,4	292	80	TAGA(CA)$_{3}$(A)$_{4}$(CA)$_{2}$CC(TG)$_{2}$(CA)$_{2}$TA(CA)$_{2}$CGC(A)$_{3}$(CA)$_{3}$TACACGCTCACGA(TA)$_{2}$//<br>//(CA)$_{3}$TACA(C)$_{3}$G
Dolphin	2	291	79	CAGA(CA)$_{3}$AATA(CA)$_{2}$CC(TG)$_{2}$(CA)$_{2}$TA(CA)$_{6}$AACACGCATA(CA)$_{10}$TA(CA)$_{3}$
	3,4	0	0	CAGA(CA)$_{3}$AATA(CA)$_{2}$CC(TG)$_{2}$(CA)$_{2}$TA(CA)$_{6}$AACACGCATA**(CA)$_{7}$CG(CA)$_{2}$**TA(CA)$_{3}$
P. Whale	1,2,3	292	79	CAGA(CA)$_{3}$AATA(CA)$_{2}$CC(TG)$_{2}$(CA)$_{2}$TA(CA)$_{6}$AACACGCATA(CA)$_{7}$CG(CA)$_{2}$TA(CA)$_{3}$
Hedgehog	1,3	322	117	(CA)$_{5}$(A)$_{3}$TA(CA)$_{3}$CC(T)$_{3}$(GC)$_{2}$AC(GC)$_{2}$GT(GC)$_{3}$A(CA)$_{2}$TA(CA)$_{15}$TATGC(A)$_{3}$(CA)$_{7}$//<br>//TA(CA)3AATA
	2,4	-2	-2	(CA)$_{5}$(A)$_{3}$TA(CA)$_{3}$CC(T)$_{3}$(GC)$_{2}$AC(GC)$_{2}$GT(GC)$_{3}$A(CA)$_{2}$TA**(CA)$_{14}$**TATGC(A)$_{3}$(CA)$_{7}$//<br>//TA(CA)$_{3}$AATA
Shrew	1,2,4	281	69	CAGA(CA)$_{3}$AATA(CA)$_{2}$CC(TG)$_{2}$(CA)$_{2}$TA(CA)$_{4}$CG(A)$_{3}$(CA)$_{4}$CG(CA)$_{2}$TA(CA)$_{3}$GACA
	3	0	0	CAGA(CA)$_{3}$AATA(CA)$_{2}$CC(TG)$_{2}$(CA)$_{2}$**TACACG(CA)$_{2}$**CG(A)$_{3}$(CA)$_{4}$CG(CA)$_{2}$//<br>//TA(CA)$_{3}$GACA
Dugong	1,2,3,4	274	67	CAGA(CA)$_{3}$AATA(CA)$_{2}$CC(TG)$_{2}$(CA)$_{2}$TA(CA)$_{3}$AA(CA)$_{4}$CT(CA)$_{3}$(CG)$_{2}$CATAC(A)$_{3}$CA
Tenrec	1,2,3,4	281	64	AGA(CA)$_{4}$AA(CA)$_{3}$CG(TG)$_{2}$(CA)$_{2}$TCCAC(GC)$_{2}$(A)$_{3}$CACG(CA)$_{2}$CTCACG(CA)$_{4}$GATG
T. wallaby	1,2,3,4	281	73	C(A)$_{3}$(CA)$_{5}$(CATA)$_{2}$CCT(TG)$_{3}$(CA)$_{2}$CG(CA)$_{2}$TG(CA)$_{2}$CT(CA)$_{2}$(CG)$_{2}$(CA)$_{6}$TCAG
Quoll	1,3,4	295	89	CAGA(CA)$_{3}$TGCCT(G)$_{3}$TG(CA)$_{2}$(C)$_{5}$GCCA(G)$_{3}$CA(C)$_{3}$ACGTG(CA)$_{2}$CG(CA)$_{4}$(C)$_{3}$ACG(CGCA)$_{2}$CAC<br>G(CA)$_{4}$CC
Echidna	1,2,3,4	317	96	(CA)$_{3}$GACATACCAACG(CA)$_{2}$TA(CA)$_{4}$TGCAGGCACG(CA)$_{3}$GACACG(CA)$_{2}$C(CA)$_{2}$(CCCA)$_{2}$CA(C<br>CCACA)$_{2}$(CA)$_{3}$GAACA

C17-4243

Species	Indiv	L_{allele}	L_{micro}	Sequence
Human	1,2,3,4	311	56	(TTTC)$_{2}$(TC)$_{7}$CC(TC)$_{5}$(C)$_{4}$(TC)$_{3}$(T)$_{8}$(TC)$_{2}$
Dolphin	1,2,3,4	303		(TC)$_{2}$(T)$_{4}$(TC)$_{5}$CC(TC)$_{4}$(C)$_{4}$(TC)$_{3}$(T)$_{7}$(TC)$_{2}$
Tenrec	1,2,4	316	61	TGTC(T)$_{4}$(TC)$_{5}$CC(TC)$_{5}$(C)$_{4}$(TC)$_{3}$CC(TC)$_{4}$(T)$_{7}$(TC)$_{2}$
	3	+2	+2	TGTC(T)$_{4}$**(TC)$_{6}$**CC(TC)$_{5}$(C)$_{4}$(TC)$_{3}$CC(TC)$_{4}$(T)$_{7}$(TC)$_{2}$
Echidna	1,2,3,4	298	43	(TC)$_{2}$(T)$_{4}$(TC)$_{4}$CCTCGC(TC)$_{2}$CC(TC)$_{3}$(T)$_{6}$CTT
Platypus	1,2,3,4	298	43	(TC)$_{2}$(T)$_{4}$(TC)$_{4}$CCTTGC(TC)$_{2}$CC(TC)$_{3}$(T)$_{6}$CTT
