# Supplementary material for: Design and Implementation of Degenerate Microsatellite Primers for the Mammalian Clade
Source: PLoS One. 2011 Dec 27;6(12):e29582. doi: 10.1371/journal.pone.0029582 (PMC3246486; doi:10.1371/journal.pone.0029582)
Supplement: Information S9 — Guidelines to facilitate the identification, design, optimization and implementation of comparative microsatellite primers. (PDF) [file pone.0029582.s009.pdf]

Supporting Information 9: Guidelines to facilitate the identification, design, optimization and implementation of comparative microsatellite primers.

- Preparation of genomic DNA:
  - Use commercial DNA extraction kits to yield high quality genomic DNA. To reduce costs, regenerate extraction columns for several rounds of extraction [1];
  - Use aliquots to store DNA extracts and limit the amount of freezing-thawing cycles that may rapidly degrade DNA quality.
  - As far as possible, avoid getting DNA from external sources.
- Identification of conserved microsatellites
  - If comparative primers for wide-ranging species are searched, alignments should contain sequences available from all species included in the same lineages;
  - If comparative primers for one particular species are looked for, alignments should only contain sequences available from closely related species. Alternatively, sequences from one additional, more distant species may help designing more robust primers;
  - Average size and pure microsatellites should be preferred as they are more mutable and evolve through mutational dynamics well explained by current models of evolution [2]. The use of very long microsatellite loci raises issues of upper length constraints and homoplasy [3].
- Identification of microsatellites with potentially conserved priming sites:
  - If a limited number of initial conserved microsatellites were selected, visual assessment on the UCSC Genome Browser is a repetitive but effective approach;
  - Alternatively, a more thorough approach may be develop if many more loci need to be reviewed, e.g. writing a script to identify at least 20 bp with no more than 3 dissimilarities in both side of microsatellites.
- Comparative primer design:
  - Designing degenerate primers using PrimaClade can give relatively good transfer results across species (e.g. C2-1218, Table 2), but this strategy limits the amount of perfect annealing to target sequences, and likely reduces the quality of genotyping and sequencing results;
  - Alternatively, non-degenerate primers where weak-bond mismatches are allowed may be preferred [4], although such mismatches also lowers affinity to target sequences in species where mutations occurred.
- PCR optimization:
  - Touchdown PCRs profiles facilitate the optimization process when many primer pairs are tested, but tailored standard profiles may be preferred for individual loci to improve the annealing specificity and amplification success.
- Genotyping
  - The use of M13 primers is only advised to reduce costs when assessing microsatellite polymorphism at the population level
  - Fluorescent primers should be purchased and used when the utility of the comparative primer pair has been shown, as they reduce the amount of primer dimer, improve the consistency of amplifications and yield a higher fluorescent signal for detection on a DNA sequencer.
- Sequencing
  - Cloning should be used to improve the consistency of sequencing results and to access sequence information from heterozygous individuals;
  - When possible, primers should be redesigned to perfectly match the target sequence in the species of interest;
  - Sequencing primers should not be synthesized with M13-tails;
  - Alternative purification methods to filter plates may be required to decrease the loss of product of interest, e.g. commercial spin-column kits and touch-prep [4].

1. Siddappa NB, Avinash A, Venkatramanan M, Ranga U (2007) Regeneration of commercial nucleic acid extraction columns without the risk of carryover contamination. Biotechniques 42: 186-192.
2. Ellegren H (2004) Microsatellites: Simple sequences with complex evolution. Nature Reviews Genetics 5: 435-445.
3. Estoup A, Jarne P, Cornuet JM (2002) Homoplasy and mutation model at microsatellite loci and their consequences for population genetics analysis. Molecular Ecology 11: 1591-1604.
4. Murphy WJ, O'Brien SJ (2007) Designing and optimizing comparative anchor primers for comparative gene mapping and phylogenetic inference. Nature Protocols 2: 3022-3030.
